# A Multistrain Mathematical Model To Investigate the Role of Pyrazinamide in the Emergence of Extensively Drug-Resistant Tuberculosis

**DOI:** 10.1128/AAC.00498-16

**Published:** 2017-02-23

**Authors:** Mariam O. Fofana, Sourya Shrestha, Gwenan M. Knight, Ted Cohen, Richard G. White, Frank Cobelens, David W. Dowdy

**Affiliations:** a Department of Epidemiology, Johns Hopkins Bloomberg School of Public Health, Baltimore, Maryland, USA; b TB Modelling Group, TB Centre, Centre for the Mathematical Modelling of Infectious Diseases, London School of Hygiene and Tropical Medicine, London, United Kingdom; c Department of Epidemiology of Microbial Diseases, School of Public Health, Yale University, New Haven, Connecticut, USA; d Amsterdam Institute for Global Health and Development, Academic Medical Center, Amsterdam, Netherlands; e KNCV Tuberculosis Foundation, The Hague, Netherlands; f Center for Tuberculosis Research, Johns Hopkins University School of Medicine, Baltimore, Maryland, USA

**Keywords:** pyrazinamide, tuberculosis, antimicrobial combinations, mathematical modeling, multidrug resistance

## Abstract

Several infectious diseases of global importance—e.g., HIV infection and tuberculosis (TB)—require prolonged treatment with combination antimicrobial regimens typically involving high-potency core agents coupled with additional companion drugs that protect against the *de novo* emergence of mutations conferring resistance to the core agents. Often, the most effective (or least toxic) companion agents are reused in sequential (first-line, second-line, etc.) regimens. We used a multistrain model of Mycobacterium tuberculosis transmission in Southeast Asia to investigate how this practice might facilitate the emergence of extensive drug resistance, i.e., resistance to multiple core agents. We calibrated this model to regional TB and drug resistance data using an approximate Bayesian computational approach. We report the proportion of data-consistent simulations in which the prevalence of pre-extensively drug-resistant (pre-XDR) TB—defined as resistance to both first-line and second-line core agents (rifampin and fluoroquinolones)—exceeds predefined acceptability thresholds (1 to 2 cases per 100,000 population by 2035). The use of pyrazinamide (the most effective companion agent) in both first-line and second-line regimens increased the proportion of simulations in which the prevalence exceeded the pre-XDR acceptability threshold by 7-fold compared to a scenario in which patients with pyrazinamide-resistant TB received an alternative drug. Model parameters related to the emergence and transmission of pyrazinamide-resistant TB and resistance amplification were among those that were the most strongly correlated with the projected pre-XDR prevalence, indicating that pyrazinamide resistance acquired during first-line treatment subsequently promotes amplification to pre-XDR TB under pyrazinamide-containing second-line treatment. These findings suggest that the appropriate use of companion drugs may be critical to preventing the emergence of strains resistant to multiple core agents.

## INTRODUCTION

Antimicrobial resistance has recently been labeled “a problem so serious that it threatens the achievements of modern medicine” (1). Concerns regarding the emergence of drug resistance in the early antimicrobial era, along with the prospect of improving clinical outcomes, led to a shift from monotherapy to combination treatment for infections of global importance, including HIV infection, tuberculosis (TB), and malaria, but the success of combination antimicrobial therapy is increasingly threatened by the rise of multidrug resistance (2–5). Combination regimens often rely on the use of highly effective core drugs that have low toxicity, high microbicidal activity, and/or a high barrier to resistance, supplemented by companion drugs that are typically less active on their own but act to enhance the overall effectiveness of the regimen while also potentially preventing the emergence of resistance to core drugs. For example, in combination therapy against HIV, nucleoside inhibitors often serve as companion agents to prevent resistance to the core drug classes of protease inhibitors, nonnucleoside reverse transcriptase inhibitors, and integrase inhibitors (6). These companion drugs are frequently reused in sequential treatment regimens when alternative companion agents are less effective or more toxic. For instance, due in part to its unique sterilizing activity against Mycobacterium tuberculosis bacilli, pyrazinamide (PZA) is used to augment the effectiveness of several core agents, including rifampin (RIF) in standard first-line TB treatment and fluoroquinolones (FQs) in most second-line regimens (7).

**FIG 1:**
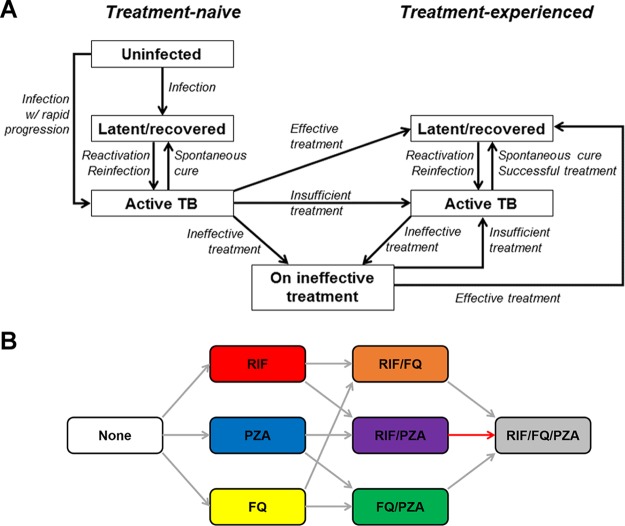
Model structure diagram. (A) The model features separate compartments for individuals who are uninfected with TB, latently infected with TB, or experiencing active disease. Individuals with TB are further distinguished on the basis of their prior treatment experience. A separate compartment exists for patients who are receiving ineffective treatment; these individuals remain ill with TB and are then initiated on a repeat course of treatment. All five TB compartments (with the exception of the uninfected compartment) are replicated for each of eight drug resistance states for a total of 41 unique compartments. Births and deaths are not shown here for simplicity. (B) Progression between drug resistance states is assumed to result only in increasing resistance. In addition to the transitions shown here, resistance to multiple drugs can be acquired within a single course of treatment. The primary mode of acquiring pre-XDR TB (defined as concomitant resistance to at least RIF and FQ) is highlighted in red and includes acquisition of resistance to PZA, a companion drug that is routinely used in both first- and second-line treatment.

**FIG 2:**
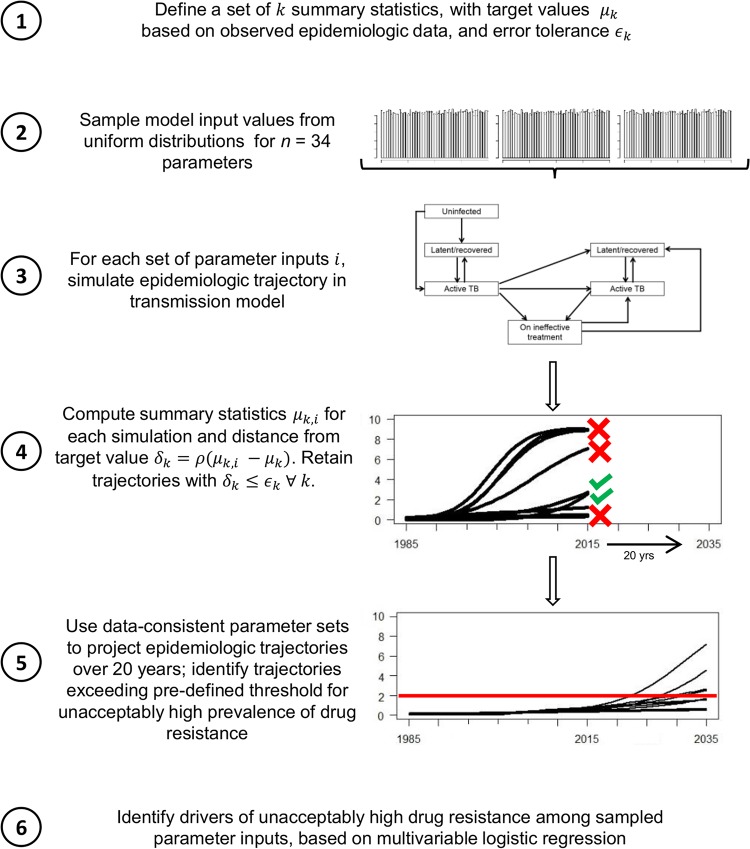
Experimental approach. Shown here is the step-by-step approach of selecting simulations that are consistent with existing epidemiologic data and projecting outcomes under those simulations, for purposes of elucidating the dynamics between strains with different patterns of resistance to multiple antimicrobial agents. ρ, distance function.

In evaluating the emergence of extensive drug resistance, research and surveillance efforts have historically focused on the role of core agents. However, the recycling of companion drugs in sequential treatment regimens may play a critical and underrecognized role in the emergence of resistance to the core agents. This is the case for PZA, which is a recommended agent in standardized first- and second-line TB treatment regimens (8). If the concomitant use of PZA prevents the emergence of resistance to RIF and FQs (an unproven hypothesis but one that is consistent with the principles of combination drug therapy), PZA resistance may therefore be an important facilitator of the emergence of strains that are resistant to both RIF and FQs, which we define conventionally to be pre-extensively drug resistant (pre-XDR) TB strains. To illustrate this concept, we constructed a dynamic model of M. tuberculosis transmission which incorporates resistance to RIF, PZA, and FQs (Fig. 1). We used this model to generate a large set of simulations consistent with epidemiological data available up to 2013 (Fig. 2). We then evaluated projected levels of pre-XDR TB in 2035, assuming that the concomitant use of PZA protects against *de novo* resistance to both RIF and FQs. We compared a baseline scenario in which PZA is recycled in first- and second-line regimens to a counterfactual scenario in which PZA is replaced by a hypothetical alternative drug of equal efficacy to demonstrate how the repeated use of companion drugs can facilitate the emergence of extensively drug-resistant strains.

## RESULTS

We first attempted to calibrate the model under our baseline assumption that PZA provides protection against *de novo* resistance to concomitantly administered RIF and FQs, as well as under the alternative assumption that PZA offers no such protection. Attempts to calibrate the model without a protective effect yielded 20-fold fewer simulations that were consistent with existing epidemiologic data (47 versus 1,015 out of 100,000 sampled parameter sets), suggesting that this assumption is probably less consistent with the available data than the assumption that PZA protects against resistance to coadministered drugs. We therefore conducted all subsequent analyses assuming that PZA protects against resistance amplification.

**FIG 3:**
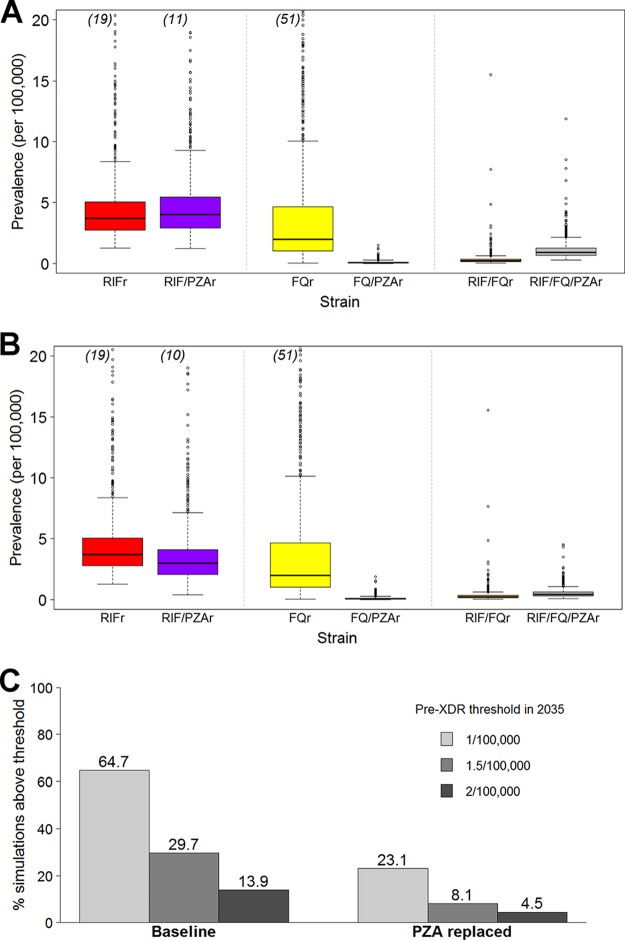
Reuse of PZA increases the projected prevalence of pre-XDR TB. Projected prevalence of RIF-resistant (RIFr), FQ-resistant (FQr), and pre-XDR (RIF- and FQ-resistant [RIF/FQr] or RIF-, FQ-, and PZA-resistant [RIF/FQ/PZAr]) TB with and without additional resistance to PZA in 2035 under the baseline (A) and PZA replacement (B) scenarios. Box plots show the median, 25th, and 75th percentile values across all data-consistent simulations. Outlier simulations with a projected pre-XDR TB prevalence of greater than 20 per 100,000 population are not shown; the numbers of such outliers, if applicable, are indicated in parentheses at the top of each box plot. (C) Proportion of data-consistent simulations in which the projected pre-XDR TB prevalence in 2035 exceeds three predefined acceptability thresholds. Replacing PZA with an alternative drug of equal efficacy among patients with PZA-resistant TB greatly reduces the proportion of trajectories in which the prevalence exceeds the pre-XDR TB acceptability threshold in 2035.

Across the 1,015 simulations consistent with epidemiologic data (assuming a protective effect of PZA on acquired resistance), the median projected prevalence of pre-XDR TB in 2015 was 0.64 per 100,000 population (interquartile range [IQR], 0.51 to 0.79 per 100,000 population). The proportion of RIF-resistant strains in 2035 that harbored additional resistance to PZA was greater in the baseline scenario (median, 51.7%; IQR, 43.7 to 59.5%) than in the alternative scenario, in which PZA was replaced (median, 44.7%; IQR, 36.4 to 51.3%), although the overall TB incidence was similar in both scenarios (median, 205.0 per 100,000 population [IQR, 1,886 to 222.5 per 100,000 population] in the baseline scenario versus 203.7 per 100,000 population [IQR, 187.6 to 221.1 per 100,000 population] in the PZA replacement scenario). There was an even more pronounced difference in the proportion of pre-XDR strains with additional PZA resistance (80.2% [IQR, 72.9 to 85.6%] in the baseline scenario versus 65.8% [IQR, 57.9 to 72.2%] in the PZA replacement scenario) (Fig. 3A and B). Overall, the proportion of simulations in which the pre-XDR prevalence exceeded predefined acceptability thresholds of 1, 1.5, and 2 per 100,000 population in 2035 was 64.7%, 29.7%, and 13.9%, respectively, in the baseline scenario, whereas it was 23.1%, 8.1%, and 4.5%, respectively, in the PZA replacement scenario. This corresponds to relative reductions of 64 to 73% in the proportion of simulations where the prevalence of pre-XDR TB exceeded each acceptability threshold. Similar results were obtained using a stochastic modeling framework: the proportion of simulations in which pre-XDR prevalence exceeded the acceptability thresholds by 2035 decreased from 52.1%, 35.7%, and 24.9%, respectively, in the baseline scenario to 25.1%, 13.7%, and 8.2%, respectively, in the PZA replacement scenario (see Fig. S9 in the supplemental material).

**FIG 4:**
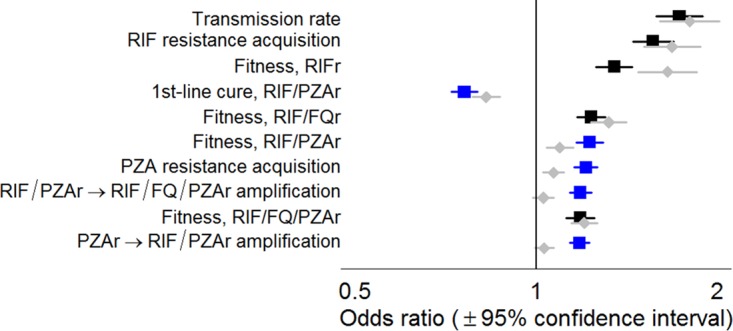
Parameters associated with a high future prevalence of pre-XDR TB. Leading drivers of future pre-XDR TB prevalence, as assessed by logistic regression on the odds of the primary outcome, namely, exceeding a predefined acceptability threshold of 1 case per 100,000 population in 2035, comparing baseline conditions (blue and black squares) to the alternative scenario in which PZA is replaced (gray diamonds). Odds ratios reflect the change in the primary outcome associated with an increase of 1/10 of a standard deviation in the independent variable. Parameters related to strains resistant to PZA only (PZAr) or resistant to both RIF and PZA (RIF/PZAr) are highlighted in blue. As an example of scale, 1/10 of a standard deviation corresponds to absolute changes of 0.5% in the probability of acquiring RIF resistance in a single course of treatment, 6% in the transmission fitness of RIF- and PZA-resistant strains, or 5% in the probability of cure for RIF- and PZA-resistant strains on the first-line regimen.

We used multivariable sensitivity analysis to investigate those parameters that were the most closely associated with the emergence of pre-XDR TB at a prevalence of 1 case per 100,000 population by 2035 (Fig. 4). Five of the 10 most influential parameters involved PZA; these included the probability of cure for RIF- and PZA-resistant TB, the transmission fitness of strains resistant to both RIF and PZA, and the probabilities of acquiring PZA resistance and subsequently developing additional resistance (Fig. 4). Under the PZA replacement scenario, the odds ratios (ORs) associated with the probabilities of acquiring PZA resistance and subsequent resistance amplification were attenuated toward a null effect (i.e., OR = 1). Sensitivity analyses varying the threshold to 1.5 and 2 pre-XDR cases per 100,000 population yielded similar findings, as did alternative analyses using partial rank correlation coefficients (Fig. S6).

**FIG 5:**
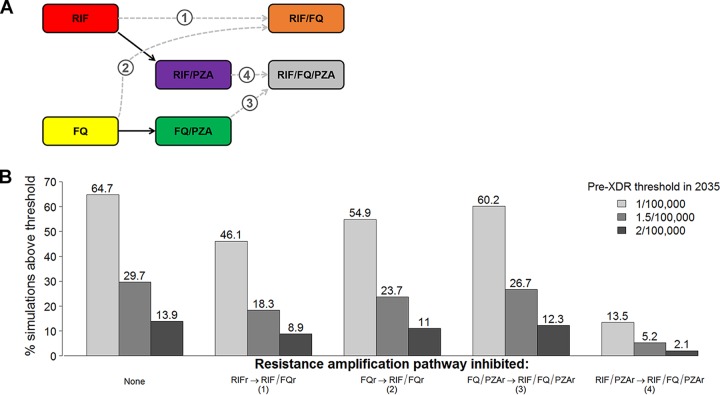
Sequential acquisition of resistance and emergence of pre-XDR TB. (A) Pathways from RIF and FQ resistance, with and without additional PZA resistance. We demonstrate that, when PZA prevents the development of resistance to RIF and FQs, the primary pathway to developing pre-XDR TB goes through an intermediate step that includes resistance to both RIF and PZA (RIF/PZAr, arrow 4), rather than directly from RIF or FQ resistance (arrows 1 and 2). (B) Proportion of data-consistent simulations in which the projected pre-XDR TB prevalence in 2035 exceeds various acceptability thresholds, after blocking specific pathways of resistance acquisition. Blocking the progression from combined RIF and PZA resistance to RIF, FQ, and PZA resistance (corresponding to arrow 4 in panel A) greatly reduces the proportion of trajectories in which the prevalence exceeds the pre-XDR TB acceptability threshold in 2035, as shown in the rightmost bars. In contrast, blocking resistance amplification directly from strains that are RIF or FQ monoresistant results in a minimal change from the baseline scenario.

Finally, we evaluated model scenarios in which specific steps in the progression to pre-XDR TB were inhibited, reflecting the potential effect of tailored therapy for patients diagnosed with PZA-resistant TB (Fig. 5). In these analyses, we found that the acquisition of FQ resistance among strains already dually resistant to RIF and PZA was a key step in the development of pre-XDR TB. Blocking this single step in resistance amplification (i.e., allowing pre-XDR TB to emerge only from strains other than RIF- and PZA-resistant strains) reduced the proportion of simulations in which the prevalence exceeded each pre-XDR acceptability threshold by 4- to 7-fold, suggesting that dual RIF and PZA resistance is an important precursor of pre-XDR TB at the population level. In contrast, blocking the emergence of pre-XDR TB from RIF-monoresistant or FQ-monoresistant strains or from FQ- and PZA-resistant strains had a minimal effect on the projected pre-XDR prevalence in 2035.

## DISCUSSION

This novel population-level modeling framework incorporating resistance to three distinct antimicrobial drugs suggests that, when companion drugs select against *de novo* resistance mutations in combination regimens, the reuse of these drugs in both first- and second-line treatment may critically facilitate the emergence of strains that are resistant to multiple core agents. Specifically, a scenario projecting the hypothetical effect of perfect susceptibility testing for PZA and replacing PZA with another drug for patients with PZA-resistant TB dramatically reduced the proportion of data-consistent model simulations in which the projected prevalence of pre-XDR TB exceeded predefined acceptability thresholds within 20 years. Simulations in which we assumed that PZA does not apply selection pressure against concomitantly administered core agents were far less likely to match available epidemiologic data. These findings highlight the urgent importance of understanding the potential mechanisms by which PZA (and other companion drugs) enhances combination antimicrobial regimens and of expanding drug susceptibility testing and surveillance for resistance to these agents, rather than focusing such efforts on core drugs alone.

Available evidence from both laboratory and clinical studies supports the sequential acquisition of resistance in TB (9, 10). Our results suggest a similar pattern at the population level and that reusing companion drugs could promote the sequential progression to pre-XDR TB during first- and second-line treatment. Specifically, we found that the prevalence of PZA resistance was greatly increased among RIF-resistant strains and even more so among pre-XDR strains when PZA was used in both first- and second-line TB treatment. Moreover, strains resistant to both RIF and PZA featured as major precursors of pre-XDR. These results suggest that the initial acquisition of RIF or PZA resistance may allow the emergence of resistance to the other agent during first-line treatment, resulting in a large number of RIF- and PZA-resistant strains. These strains are then more likely to develop FQ resistance during second-line therapy that includes both PZA and FQs. These results are highly relevant to the deployment of standardized treatment regimens for multidrug-resistant TB prescribed without prior diagnostic testing for resistance to drugs other than RIF, a practice that may become increasingly common with the scale-up of rapid molecular testing for RIF resistance alone (11–13). In settings where resistance to PZA is common, indiscriminately starting patients on FQ- and PZA-containing standardized second-line regimens (8)—at the very time when the mycobacterial burden, and thus the incidence of spontaneous resistance-conferring mutations, is the highest—may result in the selection of bacilli resistant to other drugs in the regimen, including FQs, before the results of complete drug susceptibility testing (e.g., from culture for M. tuberculosis) are available. If PZA does indeed protect against the development of resistance to FQs during second-line therapy, consistent with our model calibration and the findings of previous empirical studies, routine rapid testing for PZA resistance among patients harboring mycobacterial bacilli with demonstrated RIF resistance would be an important means of preventing the emergence of pre-XDR TB (14, 15). This finding takes on even greater significance in the current drug development climate, as FQs and PZA are considered key agents in the development of many novel regimens for first-line treatment of TB (16, 17).

Overall, our findings highlight the importance of considering not only the interplay between individual antimicrobial drugs but also how these drugs are incorporated into sequential treatment regimens, in order to better control the spread of extensive drug resistance in the long term. Although our model is specific to TB, our insights regarding the importance of recycled companion drugs in facilitating the emergence of multiresistant pathogens may be relevant to other infectious diseases in which resistance to the current arsenal of drugs represents a major public health threat. For example, HIV is a pathogen of major global health significance in which sequential resistance to antiretroviral drugs occurs over the course of treatment (18, 19). As in our study, a previous model of the development of resistance in HIV that explicitly modeled combinations of resistance to three drug classes provided important insights into drug class-specific effects on population-level resistance trajectories (20). Furthermore, by combining a population-level transmission model with policy-relevant outcome thresholds, our study provides useful guidance to decision makers in the setting of sparse empirical data on key parameters related to drug resistance. This approach, which leverages available epidemiologic data and a mechanistic understanding of disease to shed light on future trajectories of drug resistance, can be adapted to other pathogens to inform risk prediction and disease control policies.

This model has several limitations. In seeking to optimize the balance of detail and parsimony, we made several simplifying assumptions, including restricting the model to adult pulmonary TB in an equilibrium population. As our focus was on exploring long-term epidemiologic trajectories rather than clinical outcomes, we chose to exclude forms of TB (i.e., childhood and strictly extrapulmonary disease) that, despite a significant disease burden, do not contribute significantly to transmission. Similarly, we chose the Southeast Asia region, where HIV is not a major driver of the TB epidemic (21), because Southeast Asia currently has higher levels of TB drug resistance. Future adaptations of this model could evaluate different epidemiologic settings, including those in which TB is driven by HIV and those (e.g., the former Soviet Union) with a long history of drug-resistant TB that may reflect high rates of transmission of drug-resistant TB in congregate living settings (e.g., prisons). We limited our model to three key drugs for simplicity, as the addition of additional drugs creates exponentially increasing complexity. As we used a simple acceptance/rejection algorithm to select plausible parameter sets, our results should not be interpreted as probabilistic projections of future TB epidemiology. Rather, our approach allowed us to explore a representative range of data-consistent scenarios—akin to an epidemiologic study selecting a representative sample of the population—and benchmark those scenarios against potentially meaningful decision thresholds. This approach enables us to quantify both the key considerations and the level of uncertainty in such decisions, providing a risk management tool that can inform TB control policies without the need to project the precise future of drug-resistant TB. Our conclusions were unchanged when a stochastic modeling framework that better takes into account rare events in the emergence of drug resistance was used. Finally, in order to simplify our inferences on the acquisition and transmission fitness of drug-resistant strains and the treatment outcomes in patients infected with drug-resistant strains, we kept most other model parameters at fixed values and did not explicitly model changes in transmission fitness over time or potential epistatic effects; our projections may therefore underestimate the true level of uncertainty in future epidemiologic trajectories.

In summary, using a novel, multistrain modeling approach, we evaluated the impact of a companion drug on future trajectories of TB strains resistant to multiple core agents. This approach suggests that, if the companion agent (such as PZA) is used to augment the role of core drugs in both first-line and second-line regimens, the emergence of strains resistant to multiple core drugs may be dramatically hastened. As such, a key research priority should be to collect better data to understand how and to what degree companion drugs enhance the effectiveness of combination regimens (e.g., increase the probability of cure, protect against acquired resistance) and particularly how PZA impacts TB treatment. In the absence of such data, our results support the need for drug susceptibility testing for PZA prior to initiating second-line regimens that include PZA without a sufficient number of additional companion agents. These findings may generalize to infections caused by other microbial pathogens treated with sequential combination regimens, and they highlight an analytic approach that may become increasingly valuable for decision making in the setting of sparse data on resistance to multiple antimicrobial regimens.

## MATERIALS AND METHODS

### Approach.

Our aim was to understand the population-level dynamics of the emergence of multiple antimicrobial resistance in an infectious pathogen that is treated with combination therapy but for which empirical data on the effects of different resistance patterns are sparse. To achieve this aim, we used a mechanistic simulation of TB transmission and drug resistance to project a range of plausible epidemiologic trajectories, randomly sampling parameter values to reflect inherent uncertainty in key variables related to TB drug resistance (Fig. 1). First, we identified an outcome that could serve as a useful metric for decision making; in our primary analysis, we used the proportion of data-consistent trajectories in which the prevalence of pre-XDR TB exceeds an acceptability threshold of 1 case per 100,000 population at 20 years. We then selected epidemiologic data to which we could calibrate the model. These calibration targets, shown in Table S4 in the supplemental material, included the prevalence and incidence of TB disease from 1990 to 2013 in Southeast Asia (21, 22), which was selected as a target setting because of its high rates of TB and highly drug-resistant TB, as well as the prevalence of resistance against specific drugs for which empirical data were available. Further details of model initialization and calibration are provided in the supplemental material (23–27). For each epidemiologic calibration target, we set a tolerance range based on the degree of uncertainty around available data estimates (Table S4). We then constructed a representative set of scenarios that might be consistent with existing data by randomly sampling parameter sets using an approximate Bayesian process, retaining those sets that resulted in simulated outcomes within our tolerance ranges. We used these data-consistent parameter sets to project epidemiologic trajectories over the ensuing 20 years. These selected parameter sets are therefore not meant to represent the entirety of all possible scenarios, nor are they meant to indicate which scenarios are more likely than others; rather, they are meant as a representative sample that can be useful to inform decision making. This approach is illustrated step by step in Fig. 2.

### Mechanistic model structure.

The core structure of our model is similar to that of previous compartmental models of adult pulmonary tuberculosis, assuming a static population size, random mixing, and sequential progression through the stages of TB infection (28–30). As shown in Fig. 1, people are born in the uninfected state and can progress to latent TB infection (an asymptomatic, noninfectious state) and active pulmonary TB disease (symptomatic and infectious). Each compartment of TB infection or disease is subdivided to explicitly track resistance to eight (i.e., 2^3^) possible combinations of the three drugs considered. For any individual being treated for active TB, we assume that the treatment course will be effective, insufficient, or ineffective (defined below), with the probability of each outcome being conditional on both the pathogen's resistance profile and the drug regimen being used (see Table 2).

We assume that effective treatment is curative treatment that rapidly renders individuals noninfectious, reflecting the steep decrease in bacillary burden upon treatment initiation (31, 32). We include the possibility that some incomplete treatment courses may nonetheless be effective, reflecting the range of possible interactions between antimicrobial agents and host immune responses. Those patients who do not complete a full course of treatment and are not cured (i.e., patients who receive insufficient treatment) are assumed to remain ill and infectious. Treatment that results in early relapse is also represented in the model as insufficient.

In contrast to insufficient treatment (representing a treatment course that has curative potential but is simply not taken for a sufficient duration of time), ineffective treatment in this model represents a course that does not provide additional curative potential beyond the host's natural immune response. People on ineffective regimens remain infectious in this model, albeit at a reduced level, reflecting regimens that reduce the bacillary burden sufficiently to result in negative sputum smears but do not achieve sterilization and cure. The explicit modeling of ineffective treatment allows us to account for failing treatment regimens, which we assume last for 6 months, on average, reflecting a time point at which treatment effectiveness is commonly assessed (8). Individuals on ineffective regimens are assumed to remain symptomatic and/or test positive on follow-up evaluation (e.g., TB smear or culture), triggering the initiation of a repeat course of treatment. Repeat treatment may in turn be effective (leading to immediate transition to the latent compartment), insufficient (leading to a transition to the active TB compartment), or ineffective (leading to maintenance in the ineffective treatment state), depending on the regimen chosen and the resistance profile of the pathogen.

The model distinguishes patients who are undergoing their first course of TB treatment from those who have previously been treated, incorporating the greater prevalence of drug resistance among treatment-experienced patients. In the baseline scenario, we assume that 5% and 26% of treatment-naive and treatment-experienced patients with RIF-resistant TB, respectively, have access to a standardized second-line treatment regimen, reflecting a combination of access to drug susceptibility diagnostics and presumptive treatment, as estimated in this region (21).

### Incorporation of data.

Selected model inputs are shown in Tables 1 and 2 (see Table S3 for more details). Parameters relating to diagnosis and treatment outcomes are based on WHO data and information published in the literature. These data were incorporated in the model using logical assumptions; for instance, with the same regimen, the probability of cure for a patient with TB resistant to two drugs in the regimen cannot be greater than the probability of cure for a patient with TB resistant to just one drug (21, 33–37). We incorporate uncertainty around these baseline outcome probabilities by varying the probability of treatment failure from zero to twice the baseline value for each of the eight strains.

**TABLE 1:** Selected input parameters^*a*^

Variable description	Baseline value	Reference(s)
Protection from reinfection in latent infection state	0.5	41, 42
Proportion progressing rapidly to active TB	0.15	43
Baseline life expectancy (yr)	70	44
TB-specific mortality rate/yr	0.17	45
Probability of endogenous reactivation over the patient's lifetime	5%	46
Rate of diagnosis and treatment initiation/yr	0.69	21
Relative infectiousness of patients on ineffective treatment	0.2	47
Rate of spontaneous recovery from active TB/yr	0.17	45
% discontinuing treatment prior to completion		
First-line treatment	6	21
Second-line treatment	23	35
% experiencing early relapse		
Drug-sensitive TB	4	48, 49
RIF-resistant TB	16	50
FQ-resistant TB	12	50
PZA-resistant TB	8	14

a Additional details are provided in Table S3 in the supplemental material.

**TABLE 2:** Outcomes upon treatment completion, by resistance profile and treatment regimen^*a*^

Final drug resistance profile	Probability (%) of:			
	Cure		Early relapse after cure	
	First-line agents	Second-line agents	First-line agents	Second-line agents
Drug susceptible	89–99	—	4	—
RIF^r^	40–64	89–94	16	4
FQ^r^	89–99	—	4	—
PZA^r^	83–90	—	8	—
RIF^r^, FQ^r^	40–64	57–74	16	12
RIF^r^, PZA^r^	32–59	76–86	16	8
FQ^r^, PZA^r^	83–90	—	8	—
RIF^r^, FQ^r^, PZA^r^	32–59	47–68	16	12

a Additional details are provided in Table S1 in the supplemental material. —, not applicable, as the second-line regimen is assumed to be given only to patients with resistance to at least rifampin; RIF^r^, rifampin resistance; PZA^r^, pyrazinamide resistance; FQ^r^, fluoroquinolone resistance.

Some key parameters that lack reliable empirical estimates include (i) the reduction in transmissibility (transmission fitness) associated with each pattern of drug resistance, (ii) the probability of acquiring new antimicrobial resistance during treatment, and (iii) the effect of each resistance pattern on treatment outcomes for each combination of preexisting drug resistance profile and treatment regimen. For these parameters, we selected values for each simulation from broad and uniform prior distributions, reflecting the inherent uncertainty in the value of these parameters and allowing sufficient coverage of extreme values. Distributions for the probability of acquiring resistance while on each regimen were informed by a published meta-analysis (38), allowing the acquisition of resistance to more than one drug under the assumption of sequential acquisition, with preexisting drug resistance favoring the emergence of further resistance by reducing the number of active drugs.

### Baseline and comparison scenarios.

Using these distributions, we randomly sampled 100,000 distinct parameter sets to project trajectories and calibrate the mechanistic model as described above. We initiated simulations from a steady-state condition in the prechemotherapy era, sequentially introducing resistance to RIF, PZA, and FQ. All parameters were varied as described above in the baseline scenario. We also attempted to calibrate the model under the assumption that PZA confers no protection against *de novo* resistance to RIF or FQs—and, thus, that PZA resistance imposes no additional risk of such mutations—by setting the probability of acquiring resistance to RIF or FQs among individuals with PZA-resistant TB equal to that for patients with PZA-susceptible TB. We conducted all subsequent analyses assuming a protective effect of PZA and compared the baseline scenario to an alternative scenario in which all patients with PZA-resistant TB receive a hypothetical drug of equal efficacy (with regard to its impact on the probability of cure and relapse).

### Sensitivity and uncertainty analyses.

For each parameter set considered to be consistent with current epidemiologic data, we compared the proportion of trajectories with levels of pre-XDR TB that exceeded the 20-year prevalence acceptability threshold between the baseline scenario and the alternative scenario in which PZA is replaced by another drug. We then used multivariable logistic regression of standardized input parameter values on the expected probability of exceeding the threshold to identify the parameters (drivers) that are the most strongly correlated with this outcome, varying the acceptability threshold and also considering the partial rank correlation between inputs and pre-XDR prevalence in sensitivity analyses. We conducted additional analyses in which we blocked specific pathways of resistance amplification by setting the corresponding probabilities to zero, reflecting a hypothetical situation in which RIF and/or FQs are replaced by another drug of equal efficacy for patients with PZA-resistant TB. For all scenarios, we expressed uncertainty by providing the proportion of data-consistent simulations in which the prevalence of pre-XDR reached certain acceptability thresholds (rather than point estimates of pre-XDR TB resistance prevalence) and also the median and interquartile ranges of key intermediate outputs (e.g., the proportion of pre-XDR strains with concomitant PZA resistance) across all data-consistent simulations.

In order to assess the potential impact of stochastic events on the emergence (and potential die-out) of drug resistance, we constructed a stochastic adaptation of the model using the stochastic simulation algorithm adaptive tau method of Gillespie and colleagues (39) and replicated the analysis using this stochastic framework.

### Software.

The simulation model and all analyses were implemented using the software R (40). All the code necessary to replicate the analyses, tables, and figures presented here is available in an online repository: https://github.com/m-fofana/TB-PZA-model.git.

## Supplementary Material

Supplemental material
