# Supplementary material for: A Multistrain Mathematical Model To Investigate the Role of Pyrazinamide in the Emergence of Extensively Drug-Resistant Tuberculosis
Source: Antimicrob Agents Chemother. 2017 Feb 23;61(3):e00498-16. doi: 10.1128/AAC.00498-16 (PMC5328532; doi:10.1128/AAC.00498-16)
Supplement: Supplemental material [file supp_61_3_e00498-16__index.html]

A Multistrain Mathematical Model To Investigate the Role of Pyrazinamide in the Emergence of Extensively Drug-Resistant Tuberculosis — Supplemental material 

# A Multistrain Mathematical Model To Investigate the Role of Pyrazinamide in the Emergence of Extensively Drug-Resistant Tuberculosis

## Supplemental material

- Supplemental file 1 -

  Supplemental material

  PDF, 950K
