# Supplementary material for: A Multistrain Mathematical Model To Investigate the Role of Pyrazinamide in the Emergence of Extensively Drug-Resistant Tuberculosis
Source: Antimicrob Agents Chemother. 2017 Feb 23;61(3):e00498-16. doi: 10.1128/AAC.00498-16 (PMC5328532; doi:10.1128/AAC.00498-16)
Supplement: Supplemental material [file AAC.00498-16_zac003175928s1.pdf]

Understanding the drivers of drug resistance in tuberculosis

A multi-strain mathematical model

SUPPLEMENTAL MATERIAL

Mariam O. Fofana, Sourya Shrestha, Gwenan M. Knight, Ted Cohen, Richard G. White,

Frank Cobelens, David W. Dowdy

METHODOLOGIC DETAILS

Overview

This analysis projects trajectories of drug-resistant TB using a deterministic, dynamic transmission model of TB. Because several key parameters essential to modeling TB drug resistance are poorly supported with empirical data, we sample input values for these parameters. This requires us to define sampling bounds for parameters relating to the probability of acquiring resistance, the relative transmission fitness, and the probabilities of treatment outcomes for every combination of resistance to three distinct drugs, and every treatment regimen modeled in the analysis.

Effects of drug resistance

Acquired resistance to TB drugs arises due to inadequate treatment that exerts selective pressure on populations of bacilli bearing resistance-conferring mutations. In contrast, primary resistance occurs when a previously uninfected person is infected by an individual with drug-resistant TB and thus develops drug-resistant TB without any prior exposure to treatment (1).

Moreover, individuals who initiate TB treatment with pre-existing resistance are even more susceptible to developing additional resistance-conferring mutations (resistance amplification) compared to those with drug-susceptible disease (1). We assume that genetic mutations conferring drug resistance arise randomly in a bacterial population that is sufficiently large, that is, before treatment or very early in the course of treatment. Once such mutations occur, selection pressure exerted by inadequate treatment allows the mutant bacilli to multiply, resulting in clinical resistance to one or more drugs. With fewer fully effective drugs in their regimen, patients with drug-resistant TB are less likely to be cured at the end of their treatment; they also have fewer active drugs to provide a barrier against the development of further resistance during treatment (2, 3). The final outcome of treatment is conditional on both (1) the chosen treatment regimen and (2) the final resistance state (Figure S1).

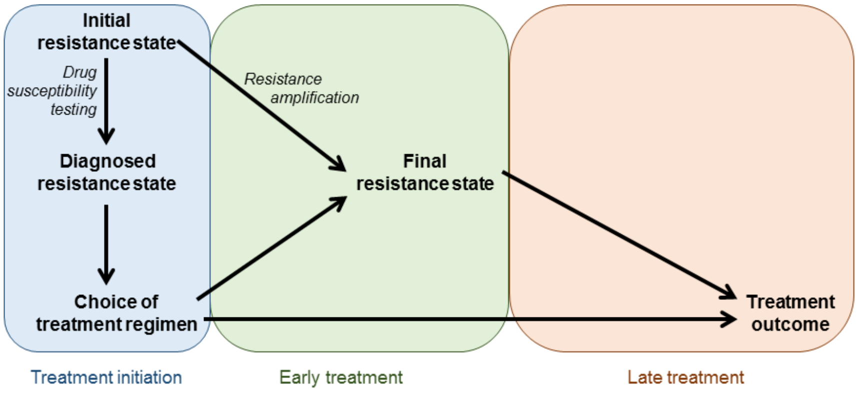

Figure S1: Effect of drug resistance and regimen choice on treatment outcomes

Regimen choice

In this analysis patients can receive any of three treatment regimens, depending on previous treatment history and available drug resistance diagnostics. The first is the standard first-line regimen consisting of isoniazid (INH), rifampin (RIF), pyrazinamide (PZA) and ethambutol (EMB) for 6 months, abbreviated as HRZE. Patients with previous TB treatment history may be prescribed a "Category II" regimen that includes the same drugs as the first-line regimen, with one additional drug, and is given for 8 months; we assume that this regimen is no more efficacious than the first-line regimen but has lower probability of completion (83% vs. 94%) (4). Patients who are identified as having RIF-resistant TB are offered a standardized second-line regimen, abbreviated as STR, consisting of a fluoroquinolone (FQ), PZA, EMB, an injectable aminoglycoside, and ethionamide, as is common practice in Southeast Asia and other settings (5-7). This regimen is given for 18-24 months, with even poorer treatment completion (77%) (8).

Regimen choice depends on the drug susceptibility diagnostic and treatment algorithm. Our model inputs for the probability of access to second-line treatment reflect not only availability of drug susceptibility testing (DST) in Southeast Asia but also other avenues to treatment. For instance, many patients are prescribed second-line treatment on the basis of clinical suspicion (e.g, failure of previous TB treatment, known contact with MDR-TB case) (4). At baseline, 5% of treatment-naïve patients and 26% of treatment-experienced patients have access to the standardized second-line regimen. The model allows for DST for RIF, FQ and PZA, with differing levels of access for treatment-naïve patients, patients who have previously failed TB treatment, and other patients with recurrent TB. The sensitivity values for resistance to each drug (RIF: 98%; FQ: 93%; PZA: 80%) reflect the state of current molecular diagnostics and can be altered to investigate hypothetical new testing technologies with improved characteristics (9-13). We assume that only detection of RIF sensitivity is available, consistent with current availability of DST in Southeast Asia (14). We assume 100% specificity for simplicity. Thus, all patients without RIF resistance (drug-susceptible or resistant to PZA and/or FQ) receive the first-line or Category II regimen, depending on previous treatment experience. For patients with any RIF resistance, the proportion receiving the standardized second-line regimen is computed as the product of the probability of access to DST and the sensitivity of RIF resistance detection.

Treatment outcomes

Although we do not explicitly model resistance to INH, empirical data indicate that most TB strains resistant to RIF are also resistant to INH (15). We therefore assume that RIF resistance in the model includes underlying resistance to INH and reflect this assumption in our treatment outcome probabilities. Table S1 lists the data sources related to the probability of cure vs. failure based on drug resistance and choice of treatment regimen. These data are sufficient to define a baseline probability of cure for every combination of resistance and treatment regimen considered in the model (Table 2). We allow for uncertainty around these outcome probabilities by randomly varying the probability of treatment failure in each simulation by a multiplicative factor of 0.75-1.25 for drug-resistant strains, or 0.5-5 for drug-susceptible TB. We apply

additional constraints in the sampling procedure to ensure that resistance to any given drug in a regimen results in poorer treatment outcomes compared to strains that do not harbor resistance to that drug. For example, TB resistant to both RIF and PZA will have poorer treatment outcomes than both RIF-resistant/PZA-susceptible and PZA-resistant/RIF-susceptible TB.

Table S1: Data sources for outcomes upon treatment completion

Outcome	Drug resistance	Value	References
First-line treatment			
Cure	Drug-susceptible	98%	(16)
Failure		2%	
Relative risk of cure vs. DS-TB	RIF	0.53	(16)
	PZA	0.86	(17)
Individualized second-line treatment			
Cure	RIF	91%	(18)
Failure		9%	
Absolute reduction in probability of cure vs. RIF resistance	RIF/FQ	16%	(19)
	RIF/PZA	0	Assumed
Standardized second-line treatment			
Absolute reduction in probability of cure vs. individualized regimen	RIF	0	Assumed
	RIF/FQ	10%	(18)

A small proportion of patients who are seemingly cured of TB at the completion of their treatment course will nevertheless experience recurrence soon thereafter (relapse). Based on published data from Southeast Asia, we set the probability of relapse for patients with drug-susceptible TB at 4%, with the remainder of patients experiencing stable cure. We apply a relative risk of 4, 3, and 2 respectively, for patients with resistance to RIF, FQ or PZA, compared to those with drug-susceptible TB, for the first-line treatment regimen (20, 21). For strains with resistance to multiple drugs, we apply the highest applicable relative risk (e.g., for a strain resistant to both RIF and PZA, we apply a relative risk of 4, for a final relapse probability of 16%). For patients receiving a second-line regimen (which does not contain RIF), we use the same principles but assume that RIF resistance has no effect on the probability of relapse; thus, we define the probabilities of relapse based only on resistance to FQ and PZA (e.g., for a strain resistant to both RIF and PZA, we apply the PZA relative risk of 2, for a final relapse probability of 8%).

Some patients recover from active TB even without completing a full course of treatment. Thus, as illustrated in Figure S2, the overall probability of recovering from active TB reflects patients who achieve a stable cure after completing treatment, as well as patients who recover despite discontinuing treatment (shown in blue). The overall probability of receiving an ineffective treatment regimen reflects patients who complete their treatment but remain infectious/symptomatic upon completion, thus prompting an immediate repeat course of

treatment (shown in gray). The overall probability of remaining infectious with active TB reflects patients who are initially thought to be cured upon treatment completion but subsequently experience relapse, as well as patients who remain infectious due to incomplete treatment (shown in red). Each of these outcomes is conditional on the final drug resistance profile j and the choice of treatment regimen k, with $c_{j|k}$, $\phi_{j|k}$, and $\sigma_{j|k}$ denoting the conditional probabilities of cure/recovery, ineffective treatment, and continued active TB, respectively, such that $c_{j|k} + \phi_{j|k} + \sigma_{j|k} = 1$.

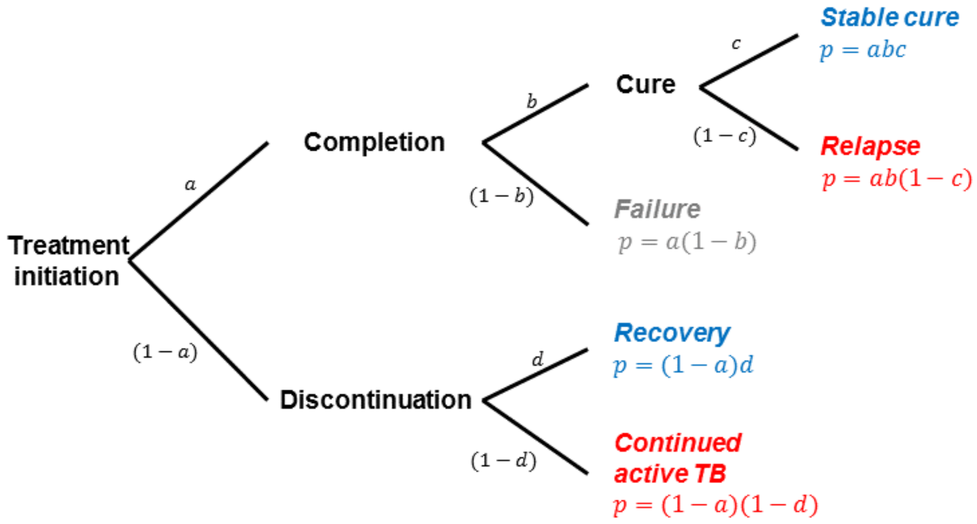

Figure S2: Treatment outcome probabilities

Resistance acquisition

Sampling bounds for the probability of acquiring resistance during a single course of treatment are based on a published meta-analysis (22). This study reported probabilities of resistance amplification of 0.008 [95% confidence interval 0.005-0.01] and 0.14 [0.09-0.2] among patients whose TB was drug-susceptible and drug-resistant at baseline, respectively. We therefore set the bounds for the probability of resistance amplification to 0-2% for patients with no pre-existing resistance to any drug in their treatment regimen, and 0-25% for patient with pre-existing resistance to one or more drugs in their treatment regimen. Resistance can only be acquired to a drug that is included in a patient's treatment regimen. However, because TB is frequently misdiagnosed as bacterial pneumonia, which is commonly treated with fluoroquinolones, we allow for some probability (0-1%) of acquiring resistance to FQ for treatment-naïve patients on the HRZE regimen. We vary this probability by 1- to 5-fold for previously treated patients, who are more likely to have been exposed to fluoroquinolones.

We assume that increasing levels of pre-existing resistance can only increase the probability of resistance amplification during treatment. For example, the probability of resistance amplification for a TB strain with pre-existing resistance to RIF and PZA must be equal to or greater than the probability for a TB strain with pre-existing resistance to RIF alone or PZA

alone. Pre-existing resistance to a drug that is not included in the treatment regimen has no effect. For example, the probability of resistance amplification under treatment with the standardized second-line regimen is the same for fully drug-susceptible strains and RIF-resistant strains, as RIF is not included in this regimen. Using the above principles and assumptions, we derive sampling bounds for each possible change in resistance profile and each treatment regimen, as shown in Table S2.

Table S2: Upper sampling bounds, probability of resistance acquisition during treatment

Initial resistance	Acquired resistance	HRZE/ Category II	Standardized 2nd-line	Additional sampling constraints
None	RIF	2%	0	
None	FQ	1%	N/A	
None	PZA	2%	N/A	
RIF	RIF/FQ	1%	2%	*Must be equal to or greater than probability of DS→FQ*
RIF	RIF/PZA	25%	2%	*Must be equal to or greater than probability of DS→PZA*
FQ	RIF/FQ	2% *	0	*Must be equal to or greater than probability of DS→RIF*
FQ	FQ/PZA	2% *	N/A	*Must be equal to or greater than probability of DS→PZA*
PZA	RIF/PZA	25%	0	*Must be equal to or greater than probability of DS→RIF*
PZA	FQ/PZA	1%	N/A	*Must be equal to or greater than probability of DS→FQ*
RIF/FQ	RIF/FQ/PZA	25% **	25%	*Must be equal to or greater than probability of RIF→RIF/PZA and FQ→FQ/PZA*
RIF/PZA	RIF/FQ/PZA	1%	25%	*Must be equal to or greater than probability of RIF→RIF/FQ and PZA→FQ/PZA*
FQ/PZA	RIF/FQ/PZA	25% †	0	*Must be equal to or greater than probability of FQ→RIF/FQ and PZA→RIF/PZA*

DS: drug-susceptible; N/A: not applicable as 2nd-line treatment only available for RIF-resistant TB
** Set equal to probability of amplification from drug-susceptible state*
*** Set equal to probability of RIF→RIF/PZA*
† Set equal to probability of PZA→RIF/PZA

We also allow for the acquisition of resistance to more than one drug in a single course of treatment. If TB bacilli acquire resistance to one drug, they then have an increased probability of acquiring resistance to a second drug within the same treatment course. We therefore assume sequential acquisition of resistance. For example, resistance can arise to drug A first, followed by drug B, or it could arise to drug B first, followed by drug A. If α_A and α_B represent the probabilities of acquiring resistance to drug A and drug B respectively in a drug-susceptible state

on a given treatment regimen, and $\alpha_{A|B}$ and $\alpha_{B|A}$ represent the probabilities of acquiring resistance to drugs A and B given pre-existing resistance to drugs B and A, respectively, then the probability of acquiring resistance to both drugs in a single treatment course is computed as: $\alpha_{AB} = \alpha_A \alpha_{B|A} + \alpha_B \alpha_{A|B}$. We can thus define the complete set of probabilities $\eta_{i,j|k}$ and $\eta^R_{i,j|k}$ for transitions from resistance state i to resistance state j, conditional on treatment regimen k, for treatment-naïve and treatment-experienced patients respectively.

Transmission fitness

We assume that even TB strains with resistance to multiple drugs are at least 50% as transmissible as drug-susceptible TB. This lower bound is supported by laboratory data estimating the fitness cost of specific drug resistance-conferring mutations in competitive growth assays (23). Although these laboratory assays are not necessarily indicative of the relative transmissibility of these TB strains at the population level, which is more difficult to assess, they do provide a reasonable bound for possible values. Laboratory data also suggest that many resistant strains are nearly as fit as drug-susceptible TB, although resistance to RIF is associated with greater costs, and MDR TB strains are known to be less transmissible (24, 25). We therefore set the bounds for the relative transmission fitness of TB strains resistant to PZA alone or FQ alone to 0.75-1, and the relative fitness of RIF-resistant strains to 0.5-1. For strains harboring resistance to multiple drugs, we set the lower bound of transmission fitness at 0.5; we further assume that their relative transmission fitness can be no greater than that of strains with less resistance. Thus, if f_A and f_B represent the relative transmission fitness of strains resistant to drug A and drug B respectively, a strain resistant to both drugs has fitness $f_{AB} \leq \min(f_A, f_B)$.

Our model allows for individuals in the latent (i.e., asymptomatic, uninfectious) TB state to become super-infected with a different strain of TB and to subsequently develop active (infectious) disease with one of the two strains (26). We assign the probability of active disease developing with one strain vs. the other based on each strains' transmission fitness values. Thus, in an individual latently infected with a strain i (fitness f_i) who becomes exposed to strain $j \neq i$ (fitness f_j), the superinfecting strain j will become dominant with probability $\zeta_{i,j} = f_j/(f_i + f_j)$. For individuals who are reinfected with the same strain (i.e., $i = j$), $\zeta_{i,i} = 1$.

Emergence of resistance and transmission

We randomly sample the time of emergence of resistance to RIF and PZA (t_1; 10 to 40 years in the past), and FQ(t_2; 10 to 30 years in the past, but after the emergence of RIF/PZA resistance). These sampling bounds reflect the timing of availability of the HRZE regimen and fluoroquinolones. The variation in the time of emergence of drug resistance partly accounts for strains that have only begun to circulate in more recent years and have variable transmission fitness due to compensatory mutations that have accumulated over time. Once the time of resistance emergence is reached in a given simulation, the probabilities of resistance acquisition are scaled up linearly over 5 years, reflecting gradual scale-up of the regimen. After setting the

sampling bounds for all of the parameters described above, we use the midpoint of each sampling range as a baseline value to calibrate the sampling range of the transmission parameter (β_0) to achieve the desired incidence and prevalence values in 2013. Based on this procedure, we set the sampling bounds for the transmission parameter at 12 ±4.

Table S3: Model input parameters

**Parameter**	**Description**	**Value/ sampling range**	**Reference(s)**
β_0	Baseline transmission rate per person-year	8-16	Calibrated
p	Proportion progressing rapidly to active TB	0.15	(27)
ψ	Rate of endogenous reactivation from latent to active TB, per year	0.007	(28)
ω_A	Baseline rate of diagnosis and treatment initiation, per year	0.69	(4)
ω_F	Rate of repeat treatment initiation for patients on ineffective treatment per year	2	(29)
ϵ	Relative susceptibility to reinfection among individuals with previous TB exposure	0.5	(30, 31)
r	Relative infectiousness of patients on ineffective treatment	0.2	(32)
h	Rate of spontaneous recovery from active TB, per year	0.17	(33)
μ_0	Baseline mortality rate, per year	1/70	(34)
μ_{TB}	TB-specific mortality rate, per year	0.17	(33)
Transmission fitness and resistance acquisition			
f_i	Relative transmission fitness, strain i	See details in "Transmission fitness" section	(23-26)
$\eta_{i,j\|k}$<br>$\eta^R_{i,j\|k}$	Probability of acquiring resistance per treatment course, from strain i to strain j, conditional on treatment regimen k, treatment-naïve or treatment-experienced	See details in "Resistance acquisition" section	(22)
m^R	Relative risk of FQ resistance acquisition on HRZE, treatment-experienced vs. treatment-naïve	1-5	Assumed
Treatment outcomes			
$x_{i,k}$<br>$x^R_{i,k}$	Proportion receiving regimen k among those with TB strain i, treatment-naïve and treatment-experienced	See details in "Regimen choice" section	(4, 11, 12, 14)
$c_{i\|k}$	Probability of cure/recovery with strain i and treatment regimen k	See details in "Treatment outcomes" section	(16-19)
$\sigma_{i\|k}$	Probability of remaining in active TB state with strain i and treatment regimen k		
$\phi_{i\|k}$	Probability of ineffective treatment with strain i and treatment regimen k		

State transitions and model equations

- *Model state compartments*

The 6 major compartments in the model reflect the natural history and treatment of tuberculosis, as shown in Figure 1, with the "R" subscript denoting patients with previous treatment experience:

- U: Uninfected
- L & L^R: Latent infection/recovered from active TB
- A & A^R: Active TB disease
- F: On ineffective (failing) treatment

All compartments (with the exception of the Uninfected compartment) are further subdivided into 8 possible resistance profiles according the infecting TB strain, as denoted by the subscript i, where $1 \le i \le 8$. Thus,

- $L = \sum_{i=1}^{8} L_i$; $L^R = \sum_{i=1}^{8} L_i^R$
- $A = \sum_{i=1}^{8} A_i$; $A^R = \sum_{i=1}^{8} A_i^R$
- $F = \sum_{i=1}^{8} F_i$

The total population is thus computed as $N = U + \sum_{i=1}^{8}(L_i + L_i^R + A_i + A_i^R + F_i)$.

- *Force of infection*

We define λ_i as the strain-specific force of infection, which depends on the prevalence of each strain in the population as well as the relative transmission fitness. Thus, for any strain i, with transmission fitness f_i:

$$\lambda_i = \beta_0 f_i I_i / N$$

$I_i = A_i + A_i^R + rF_i$, reflecting the total number of individuals with active TB caused by strain i, and accounting for the reduced transmission among individuals on ineffective treatment (compartments F_i).

- *Initial infection*

A proportion p of individuals who initially become infected with strain i progress immediately to active TB, with the remainder advancing to latent TB. Thus, the *per capita* rates of progression upon initial infection are:

- $U \to L_i$: $(1 - p)\lambda_i$
- $U \to A_i$: $p\lambda_i$

- *Spontaneous recovery*

Spontaneous recovery among both treatment-naïve and treatment experienced individuals occurs at the same rate:

- $A_i \to L_i: h$
- $A_i^R \to L_i^R: h$

- *Reactivation*

Progression from latent infection to active disease among both treatment-naïve and treatment-experienced individuals occurs at the same rate:

- $L_i \to A_i: \psi$
- $L_i^R \to A_i^R: \psi$

- *Reinfection*

Individuals latently infected with strain i can become reinfected with any other strain j, but have reduced susceptibility to infection. As in initial infection, a proportion p progress immediately to active disease. The probability $\zeta_{i,j}$ that the super-infecting strain j will become dominant is determined by the relative transmission fitness of the two strains, as described earlier.

- $L_i \to L_j: (1-p)\lambda_j \epsilon \zeta_{i,j}, i \neq j$
- $L_i^R \to L_j^R: (1-p)\lambda_j \epsilon \zeta_{i,j}, i \neq j$
- $L_i \to A_j: p\lambda_j \epsilon \zeta_{i,j}, i \neq j$
- $L_i^R \to A_j^R: p\lambda_j \epsilon \zeta_{i,j}, i \neq j$

- *Successful treatment*

Individuals exit the active TB compartments according to the baseline rate of diagnosis and treatment initiation. The probability of recovery depends on whether additional resistance is acquired during treatment, and on the probability of cure/recovery based on the final resistance state j given treatment regimen k.

- $A_i \to L_j^R: \omega_A \sum_k (x_{i,k} \eta_{i,j|k} c_{j|k}), i \leq j$
- $A_i^R \to L_j^R: \omega_A \sum_k (x_{i,k}^R \, \eta_{i,j|k}^R c_{j|k}), i \leq j$

Patients on ineffective treatment immediately begin a new course of treatment after an average of 6 months.

- $F_i \to L_j^R: \omega_F \sum_k (x_{i,k}^R \, \eta_{i,j|k}^R c_{j|k}), i \leq j$

- *Ineffective and insufficient treatment*

The rates of transition associated with ineffective and insufficient treatment are computed similarly, based on the probabilities for each treatment outcome.

- $A_i \rightarrow F_j$: $\omega_A \sum_k (x_{i,k}\eta_{i,j|k}\phi_{j|k}), i \leq j$
- $A_i^R \rightarrow F_j$: $\omega_A \sum_k (x_{i,k}^R \eta_{i,j|k}^R \phi_{j|k}), i \leq j$
- $F_i \rightarrow F_j$: $\omega_F \sum_k (x_{i,k}^R \eta_{i,j|k}^R \phi_{j|k}), i < j$

- $A_i \rightarrow A_j^R$: $\omega_A \sum_k (x_{i,k}\eta_{i,j|k}\sigma_{j|k}), i \leq j$
- $A_i^R \rightarrow A_j^R$: $\omega_A \sum_k (x_{i,k}^R \eta_{i,j|k}^R \sigma_{j|k})\ , i \leq j$
- $F_i \rightarrow A_j^R$: $\omega_F \sum_k (x_{i,k}^R \eta_{i,j|k}^R \sigma_{j|k}), i \leq j$

- *Births and deaths*

The baseline mortality rate is applied to the U and L compartments, and an increased mortality rate is applied to patients with active TB. The population is kept constant such that the number of births equals the total number of deaths, with all births occurring in the uninfected compartment:

$$\mu_0 \sum_{i=1}^{8} (L_i + L_i^R) + \ \mu_{TB} \sum_{i=1}^{8} (A_i + A_i^R + F_i)$$

The full system of ordinary differential equations in the model can thus be summarized as follows, where subscripts $i, j \in \{1, \ldots, 8\}$ denote each TB strain, and subscript $k \in \{1,2,3\}$ denotes the treatment regimen:

(1) $\frac{dU}{dt} = \mu_0 \ \sum_i (L_i + L_i^R) + \mu_{TB} \sum_i (A_i + A_i^R + F_i) - U \sum_i \lambda_i$

(2) $\frac{dL_i}{dt} = (1-p)\lambda_i U + h\, A_i + (1-p)\epsilon \sum_{j \neq i} (\lambda_i \zeta_{j,i} L_j) - \left[\epsilon \sum_{j \neq i} (\lambda_j \zeta_{i,j}) + \psi + \mu_0\right] L_i$

(3) $$\frac{dA_i}{dt} = p\ \lambda_i U + \psi L_i + p\ \epsilon \sum_{j \neq i} (\lambda_i \zeta_{j,i} L_j) - \left[\omega_A \sum_{j \geq i,k} \left(x_{i,k}\eta_{i,j|k}(c_{j|k} + \ \phi_{j|k} + \ \sigma_{j|k})\right) + h + \ \mu_{TB}\right] A_i$$

(4) $$\frac{dF_i}{dt} = \ \omega_A \left[\sum_{j \leq 2,k} (x_{j,k}\eta_{j,i|k}\phi_{i|k} A_j + x_{j,k}^R \eta_{j,i|k}^R \phi_{i|k} A_j^R)\right] + \omega_F \sum_{j<i,k} (x_{j,k}^R \eta_{j,i|k}^R \phi_{i|k} F_j) - \left[\omega_F \ \sum_{j \geq i,k} \left(x_{i,k}^R \ \eta_{i,j|k}^R (c_{j|k} + \ \sigma_{j|k})\right) + \ \omega_F \sum_{j>i,k} (x_{i,k}^R \ \eta_{i,j|k}^R \ \phi_{j|k}) + \mu_{TB}\right] F_i$$

(5) $$\frac{dL_i^R}{dt} = h\ A_i^R + \ \omega_A \sum_{j \leq i,k} (x_{j,k}\eta_{j,i|k} c_{i|k}\ A_j) + \ \omega_A \sum_{j \leq i,k} (x_{j,k}^R \eta_{j,i|k}^R c_{i|k}\ A_j^R) + \sum_{j \leq i,k} (x_{j,k}^R \eta_{j,i|k}^R c_{i|k}\ F_j) + (1-p)\epsilon \sum_{j \neq 2} (\lambda_i \zeta_{j,i} L_j) - \left[\psi + \epsilon \sum_{j \neq i} (\lambda_j \zeta_{i,j}) + \mu_0\right] L_i^R$$

(6) $$\frac{dA_i^R}{dt} = \psi L_i^R + p\ \epsilon \sum_{j \neq i} (\lambda_i \zeta_{j,i} L_j^R) + \ \omega_A \sum_{j \leq i,k} (x_{j,k}\eta_{j,i|k}\sigma_{i|k} A_j) + \omega_F \sum_{j \leq i,k} (x_{j,k}^R \eta_{j,i|k}^R \sigma_{i|k} F_j) + \ \omega_A \sum_{j \leq i,k} (x_{j,k}^R \eta_{j,i|k}^R \sigma_{i|k} A_j^R) - \left[h + \ \omega_A \sum_{j \geq i,k} \left(x_{i,k}^R \eta_{i,j|k}^R (c_{j|k} + \ \phi_{j|k})\right) + \ \omega_A \sum_{j>i,k} (x_{i,k}^R \eta_{i,j|k}^R \phi_{j|k}) + \mu_{TB}\right] A_i^R$$

Simulation selection

After generating 100,000 simulations using inputs sampled from uniform distributions with bounds as described above, we retain trajectories that are consistent with current epidemiologic data, using a procedure analogous to an approximate Bayesian computation rejection algorithm, as illustrated in Figure 2 (35). The choice of uniform prior distributions reflects inherent uncertainty about the values of these parameters. Compared to peaked distributions, uniform distributions also increase sampling from the bounds of the sampling range, thus ensuring that our sampled parameters sets include scenarios that, although unlikely, are important in evaluating the plausibility of extreme epidemiologic scenarios.

We generate simulations under two alternative assumptions: (1) that PZA provides protection against the development de novo mutations conferring resistance to RIF or FQ during treatment and (2) that PZA provides no such protection. In the baseline scenario, we sample all parameters inputs as described above. In the alternative ("no-protection") scenario, we modify the probabilities of resistance acquisition such that PZA resistance has no effect on further resistance amplification. For example, we set the resistance acquisition probabilities for PZAr → RIF/PZAr amplification equal to the sampled values for DS → RIFr amplification. Thus, the "no-protection" scenario features lower probabilities of resistance amplification among all strains with PZA resistance, compared to the baseline scenario.

Overall, 1.1% of simulations under the baseline scenario projected epidemiologic trajectories consistent with available epidemiologic data for Southeast Asia (Figures S3, S4). The proportion of trajectories meeting each of the calibration criteria is shown in Table S4. To assess the effect of this procedure, we examine the posterior distributions of our input parameters among the selected trajectories and compare them to the uniform prior distributions using the Kolmogorov-Smirnoff statistic (Table S5). Attempts to calibrate the model to Southeast Asia data under the "no-protection" scenario had a much lower yield, with only 47 of 100,000 simulations (0.05%) meeting the calibration criteria, primarily due to an inability to match the reported prevalence of RIF resistance among FQ-resistant retreatment cases (Table S4, Figure S4). Although it is also possible that our model was unable to match available data under the no-protection scenario because it cannot not fully capture the dynamics of such a system, we interpreted this incongruence of model output and epidemiologic data as indicating that the no-protection scenario is less plausibly reflective of the true effect of PZA. This interpretation is consistent with the ten-fold difference in yield of data-consistent simulations between the two scenarios, and previous empirical studies that support a protective role of PZA (21, 36). We therefore retain the data-consistent parameters generated under the baseline scenario for all subsequent analyses, and run all simulations assuming a protective effect of PZA against resistance to RIF and FQs.

Regression and correlation analyses

To identify the primary drivers of drug resistance trajectories, we first categorize each selected trajectory based on whether it results in a prevalence of drug resistance exceeding a set threshold within 20 years. We scale all of the sampled parameters to z-scores based on the empirical distribution of values among the selected trajectories. We then estimate a multivariate logistic regression model, using the z-scores as explanatory variables and report regression coefficients and odds ratios associated with a change of 0.1 standard deviation. We exclude explanatory

variables found to have excessive collinearity based on a variance inflation factor >10 in a stepwise procedure, until no such parameters remain in the model (37). Once the final model is defined, we select variables with a statistically significant regression coefficient ($p<0.05$) and rank them based on the absolute value of the coefficient. We conduct a similar analysis using partial rank correlation coefficients (PRCCs) on the original (i.e., not scaled) values for the prevalence of drug resistance.

Alternate epidemiologic settings

In order to assess the applicability of our results to settings with the highest TB burden, we selected the 100 simulations that were most representative of TB epidemiology in these countries, as well as in the Southeast Asia region, in terms of overall TB incidence and prevalence of MDR TB. For each country (India, Pakistan, Indonesia), we compared WHO data to our simulations assuming a joint Poisson likelihood function with the WHO-reported estimate as the mean. Although the absolute estimates of the proportion of trajectories resulting in a prevalence of pre-XDR TB exceeding the predefined thresholds changes, the overall findings are robust: replacing PZA with an alternative drug of similar efficacy greatly reduces the projected prevalence of pre-XDR TB (Table S5).

Stochastic model adaptation

We adapted the system of differential equations shown above to a stochastic model using the Gillespie stochastic simulation adaptive tau method, as implemented in the R package "adaptivetau" (38). We further modified the model by incorporating scale-up of MDR treatment, improvements in case detection, and decline in TB incidence reflective of trends in Southeast Asia in 1995-2013, and applied a 2% annual decrease in incidence to better reflect regional trends (39). We generated 200,000 randomly sampled values for key model inputs, as described above, and projected 1 stochastic trajectory for each parameter set, using a population size of 10 million individuals. We retained 1,751 trajectories that met our pre-defined calibration targets based on available epidemiologic data from Southeast Asia (Table S4). We used these simulations to replicate all subsequent analyses and compare our findings to those obtained using the deterministic version of the model.

Table S4: Calibration criteria

Epidemiologic criteria	Target value		References	Calibration range	Trajectories within range (%)	
	Year	*Value*			*Baseline*	*No protection*
Annual TB incidence, per 100,000	2013<br>2010<br>2005<br>2000<br>1995<br>1990	183<br>194<br>213<br>220<br>218<br>218	(4)	137-229<br>145-242<br>160-266<br>165-275<br>163-272<br>163-272	42%	42%
RIF-resistant among new cases (%)	2013	2.2%	(4)	1.1-3.3%	32%	29%
RIF-resistant among retreatment cases (%)	2013	16%	(4)	8-24%	46%	40%
RIF-resistant among retreatment cases with FQ resistance (%)	2013	25%	(40)	10-40%	16%	1.8%
RIF-resistant among retreatment cases with PZA resistance (%)	2013	55%	(41)	40-70%	45%	30%
PZA-monoresistant among new cases (%)	2013	< % RIF resistance	(42, 43)	--	86%	84%
FQ-monoresistant among new cases (%)	2013	< % RIF resistance	(44, 45)	--	99%	99%

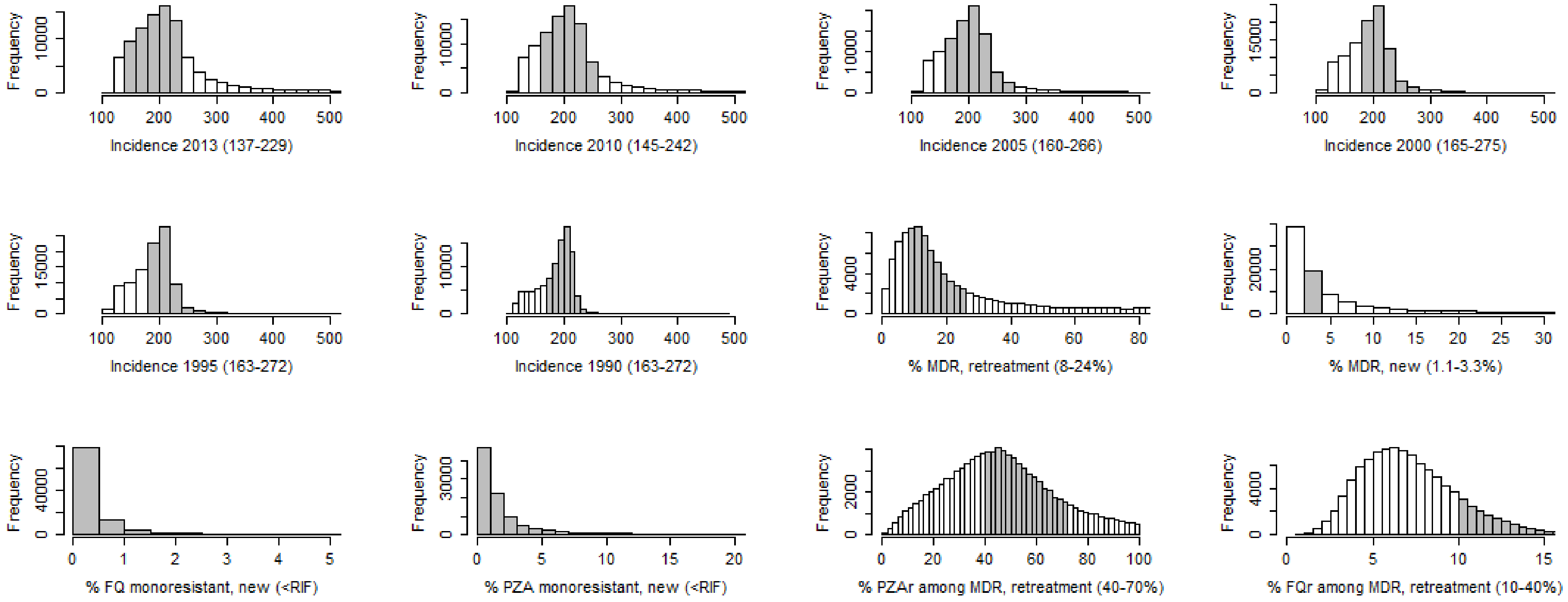

Figure S3: Distribution of calibration criteria, baseline scenario

Distribution of simulation outcomes used for model calibration, assuming a protective effect of PZA against the development of mutations conferring resistance to RIF and FQ. Values meeting the calibration criteria (shown in parentheses) are colored in gray.

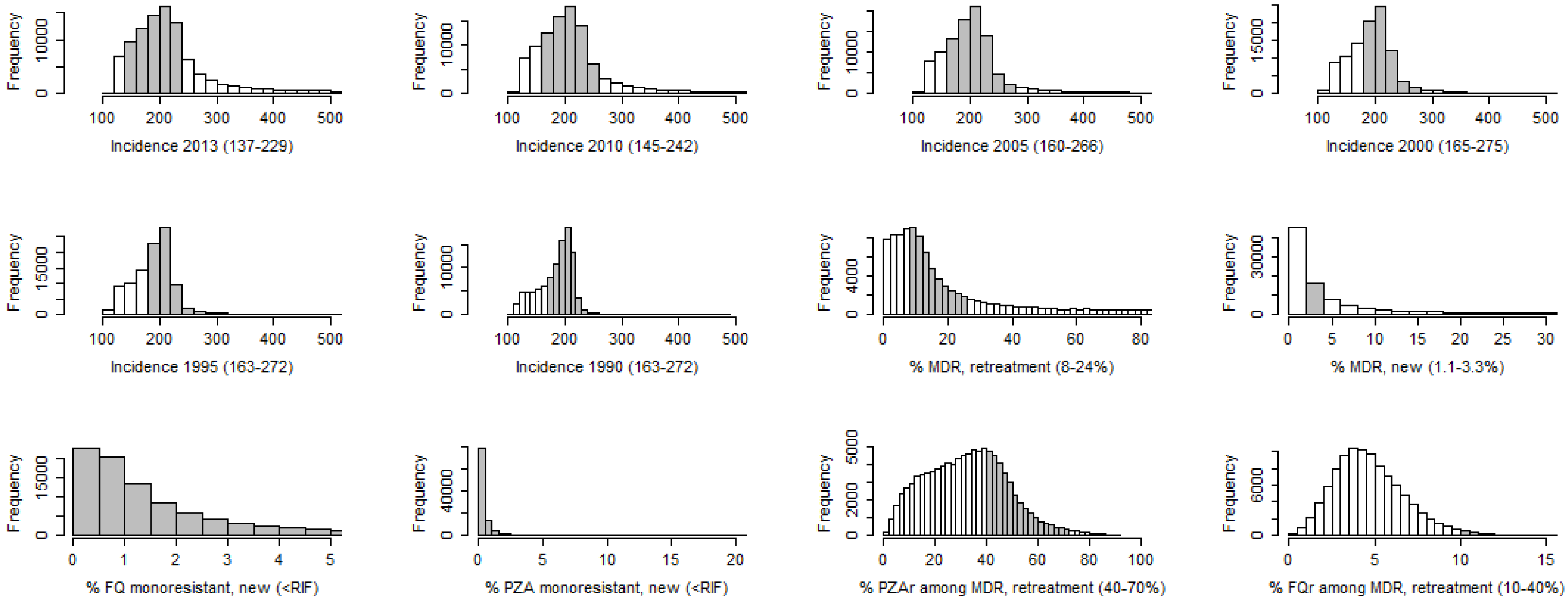

Figure S4: Distribution of calibration criteria without protection effect

Distribution of simulation outcomes used for model calibration, assuming that PZA confers no protective effect against the development of mutations conferring resistance to RIF and FQ. Values meeting the calibration criteria (shown in parentheses) are colored in gray. This assumption resulted in 20 times fewer simulations matching epidemiologic calibration criteria.

Table S5: Distribution of sampled input parameters before and after selection of simulations consistent with current epidemiology (baseline scenario)

	Sampled values			Data-consistant values			D statistic	p-value
	Median	25th %ile	75th %ile	Median	25th %ile	75th %ile		
Time of emergence of resistance								
RIF, PZA	34.864	27.459	42.444	32.681	26.615	39.278	0.120	0.000
FQ	44.652	39.445	47.997	41.610	36.813	45.512	0.210	0.000
Probability of resistance acquisition, HRZE regimen								
DS→RIFr	0.010	0.005	0.015	0.011	0.008	0.015	0.147	0.000
DS→FQr	0.005	0.002	0.008	0.006	0.004	0.008	0.174	0.000
DS→PZAr	0.010	0.005	0.015	0.010	0.005	0.015	0.019	0.849
RIFr→RIF/FQr	0.008	0.006	0.009	0.009	0.008	0.010	0.201	0.000
RIFr→RIF/PZAr	0.130	0.070	0.190	0.188	0.145	0.220	0.328	0.000
PZAr→RIF/PZAr	0.130	0.070	0.190	0.155	0.098	0.206	0.124	0.000
PZAr→FQ/PZAr	0.008	0.006	0.009	0.009	0.007	0.010	0.119	0.000
RIF/PZAr→RIF/FQ/PZAr	0.010	0.009	0.010	0.010	0.009	0.010	0.136	0.000
Probability of resistance acquisition, standardized 2nd-line regimen								
RIFr→RIF/FQr	0.010	0.005	0.015	0.011	0.006	0.016	0.080	0.000
RIFr→RIF/PZAr	0.010	0.005	0.015	0.011	0.006	0.016	0.054	0.006
RIF/FQr→RIF/FQ/PZAr	0.131	0.070	0.191	0.130	0.067	0.188	0.021	0.760
RIF/PZAr→RIF/FQ/PZAr	0.131	0.070	0.190	0.192	0.139	0.223	0.306	0.000
Transmission fitness								
RIFr	0.750	0.625	0.875	0.634	0.578	0.698	0.388	0.000
FQr	0.875	0.812	0.937	0.869	0.813	0.929	0.046	0.031
PZAr	0.875	0.813	0.938	0.818	0.782	0.866	0.291	0.000
RIF/FQr	0.589	0.532	0.678	0.561	0.524	0.620	0.163	0.000
RIF/PZAr	0.589	0.533	0.677	0.551	0.521	0.597	0.248	0.000
FQ/PZAr	0.661	0.580	0.742	0.651	0.579	0.726	0.062	0.001
RIF/FQ/PZAr	0.515	0.504	0.540	0.512	0.504	0.531	0.074	0.000
Probability of cure, HRZE regimen								
DS, FQr	0.940	0.915	0.965	0.942	0.915	0.965	0.027	0.441
RIFr, RIF/FQr	0.520	0.460	0.580	0.490	0.439	0.560	0.136	0.000
PZAr, FQ/PZAr	0.865	0.847	0.882	0.866	0.846	0.882	0.016	0.956
RIF/PZAr, RIF/FQ/PZAr	0.406	0.363	0.463	0.372	0.344	0.407	0.248	0.000
Probability of cure, standardized 2nd-line regimen								
RIFr	0.915	0.903	0.928	0.914	0.902	0.927	0.028	0.426
RIF/FQr	0.661	0.621	0.701	0.658	0.616	0.697	0.044	0.038
RIF/PZAr	0.810	0.785	0.835	0.808	0.785	0.833	0.038	0.104
RIF/FQ/PZAr	0.583	0.531	0.631	0.574	0.521	0.624	0.061	0.001
Relative risk of FQ resistance acquisition retreatment vs. new cases								
	2.998	2.009	4.001	3.933	3.090	4.539	0.280	0.000
Transmission parameter								
	12.005	10.013	13.999	11.886	10.810	13.149	0.209	0.000

SUPPLEMENTAL RESULTS

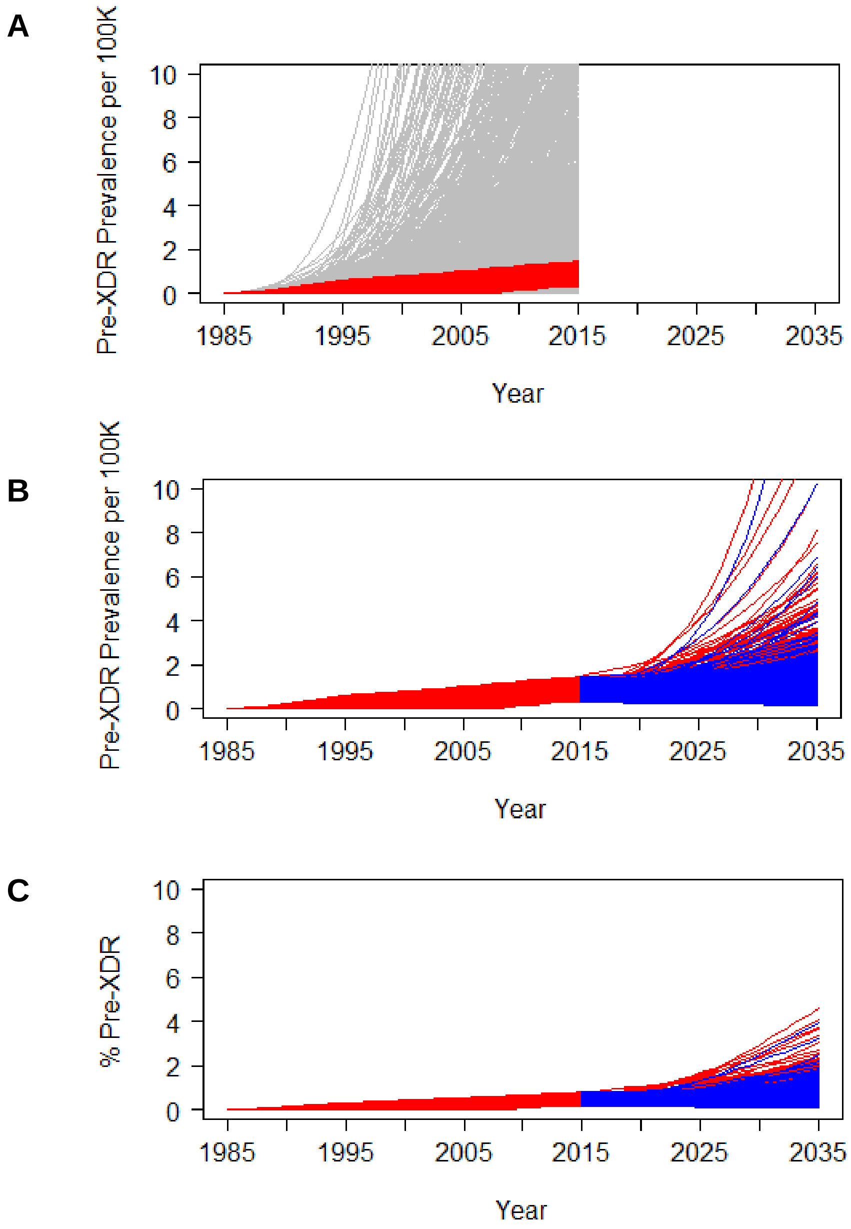

Figure S6: Projected trajectories of pre-XDR TB

A) Random subsample of generated trajectories up to 2015, shown in gray.

B-C) Even after selecting for trajectories consistent with current TB epidemiology, shown in red, the range of drug resistance prevalence (B) and the proportion of drug-resistant TB cases (C) in 2035 vary widely. Replacing PZA with an equally effective drug in the treatment regimens of patients with PZA-resistant TB greatly reduced projected levels of pre-XDR TB, as shown by the overlaid blue curves.

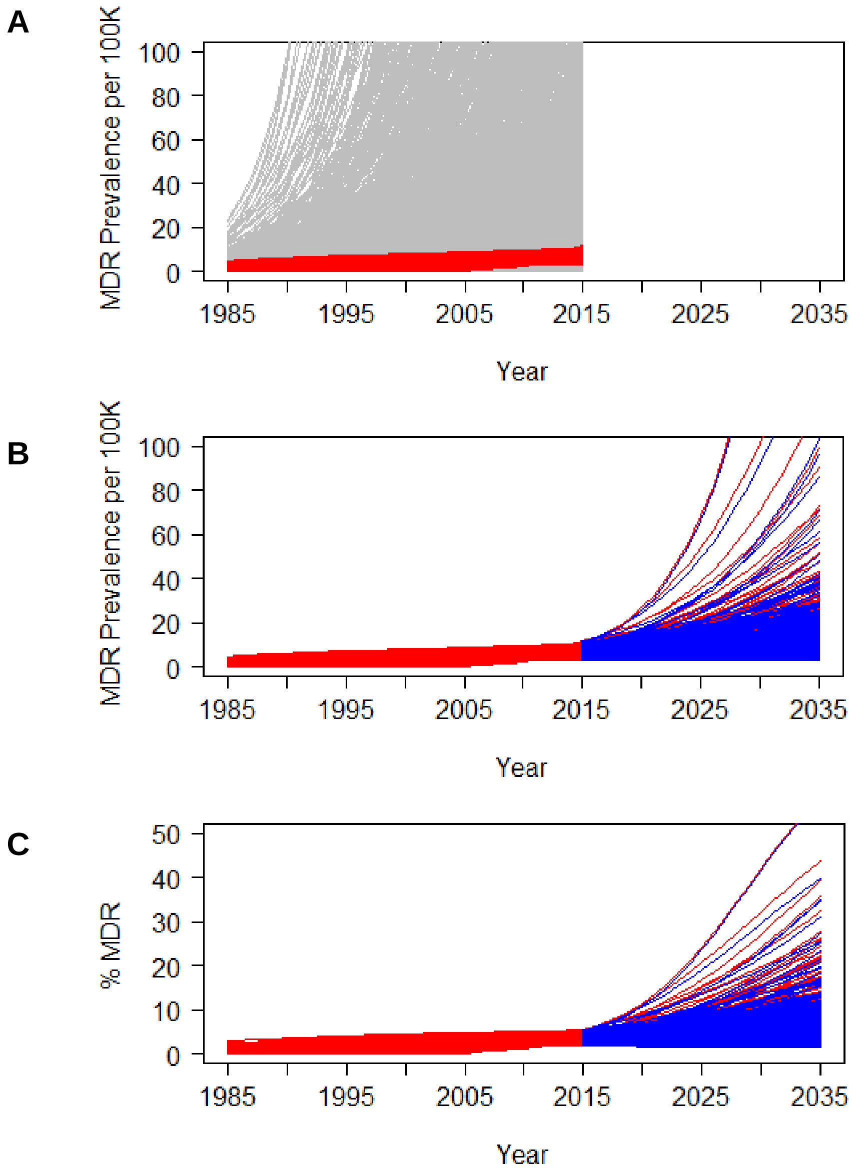

Figure S7: Projected trajectories of RIF-resistant TB

A) Random subsample of generated trajectories up to 2015, shown in gray.

B-C) Data-consistent trajectories of prevalence (B) and proportion (C) of pre-XDR TB projected to 2035, shown in red. Replacing PZA with an equally effective drug in the treatment regimens of patients with PZA-resistant TB (blue) had little impact on projected trajectories.

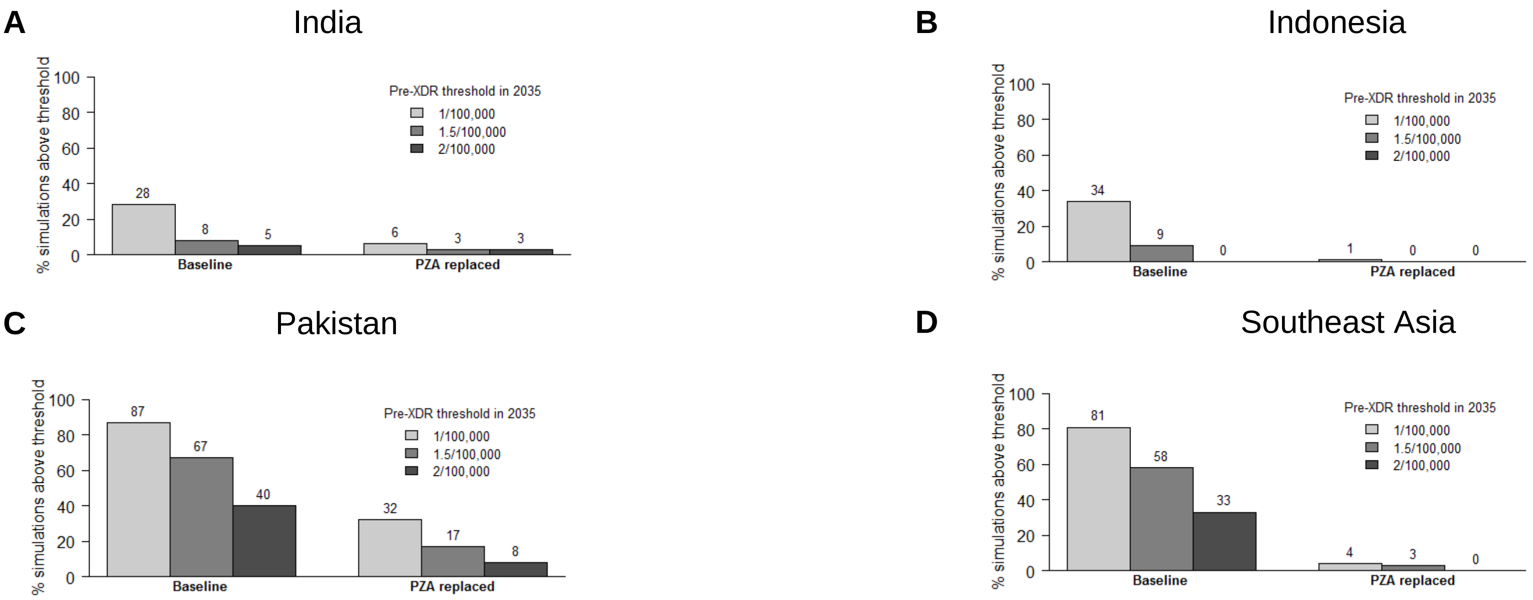

Figure S8: Impact of PZA replacement on projected prevalence of pre-XDR-TB across high TB burden settings

Proportion of data-consistent simulations in which projected pre-XDR TB prevalence in 2035 exceeds three pre-defined acceptability thresholds. We selected the 100 simulations most representative of TB epidemiology in India (A), Indonesia (B), Pakistan (C) and the Southeast Asia region (D). In all cases, replacing PZA with an alternative drug of equal efficacy among patients with PZA-resistant TB greatly reduces the proportion of trajectories exceeding the pre-XDR TB acceptability threshold in 2035.
PZA: pyrazinamide

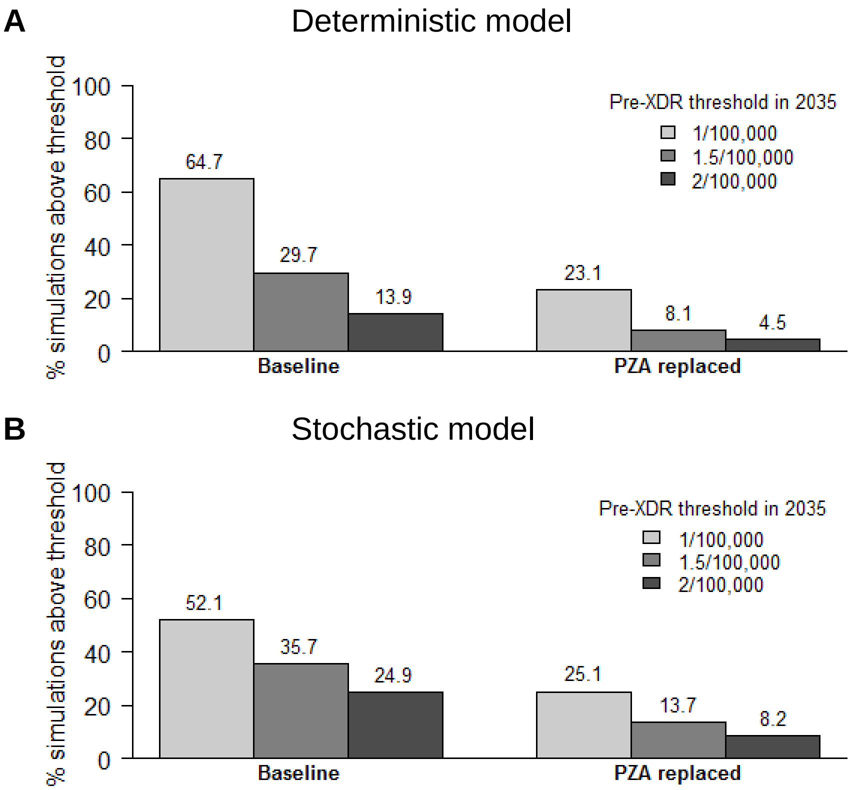

Figure S9: Impact of PZA replacement on projected prevalence of pre-XDR-TB, in deterministic vs. stochastic model

Proportion of data-consistent simulations in which projected pre-XDR TB prevalence in 2035 exceeds three pre-defined acceptability thresholds. In both the deterministic and the stochastic frameworks, replacing PZA with an alternative drug of equal efficacy among patients with PZA-resistant TB greatly reduces the proportion of trajectories exceeding the pre-XDR TB acceptability threshold in 2035.
PZA: pyrazinamide

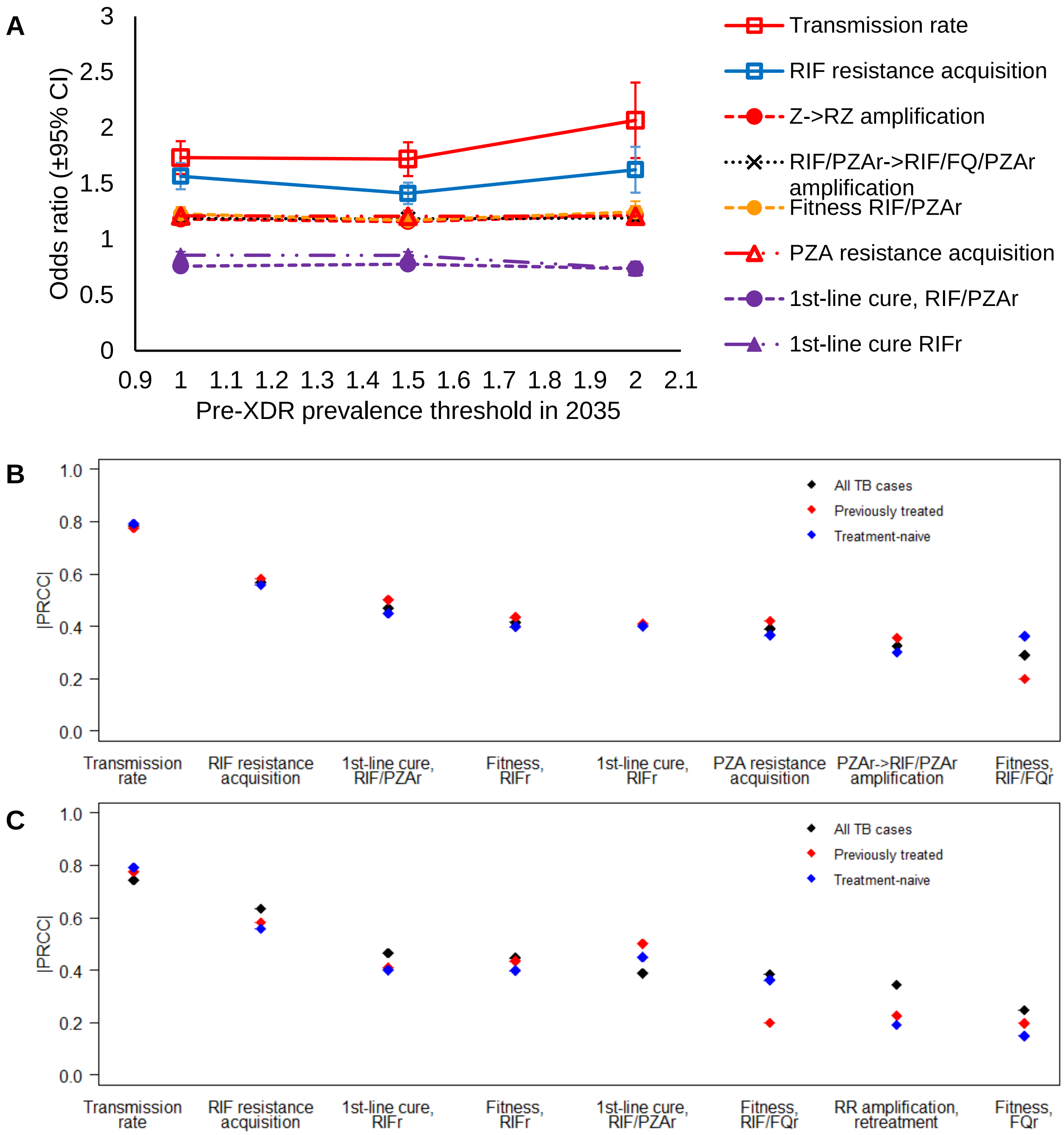

Figure S10: Factors associated with high future pre-XDR TB prevalence

A) Odds ratios (with 95% confidence intervals) for parameters most strongly correlated with prevalence of pre-XDR TB exceeding 1, 1.5, or 2 cases per 100,000 population in 2035, baseline scenario. Similar results for the baseline scenario are obtained with alternative analyses using partial rank correlation coefficients (B). In contrast, PZA-related parameters became less predictive of pre-XDR prevalence if PZA was replaced with an equivalent alternative drug for patients with PZA-resistant TB (C).

REFERENCES

1. **Zhang Y, Yew WW.** 2009. Mechanisms of drug resistance in Mycobacterium tuberculosis. Int J Tuberc Lung Dis **13:**1320-1330.
2. **Mitchison DA.** 1998. How drug resistance emerges as a result of poor compliance during short course chemotherapy for tuberculosis. Int J Tuberc Lung Dis **2:**10-15.
3. **Gillespie SH.** 2002. Evolution of drug resistance in Mycobacterium tuberculosis: clinical and molecular perspective. Antimicrob Agents Chemother **46:**267-274.
4. **World Health Organization.** 2014. Global Tuberculosis Control 2014. WHO, Geneva. http://www.who.int/tb/publications/global_report/en/. Accessed June 29, 2015.
5. **Muller B, Chihota VN, Pillay M, Klopper M, Streicher EM, Coetzee G, Trollip A, Hayes C, Bosman ME, Gey van Pittius NC, Victor TC, Gagneux S, van Helden PD, Warren RM.** 2013. Programmatically selected multidrug-resistant strains drive the emergence of extensively drug-resistant tuberculosis in South Africa. PLoS One **8:**e70919. doi:10.1371/journal.pone.0070919.
6. **Saravia JC, Appleton SC, Rich ML, Sarria t, Bayona J, Becerra MC.** 2005. Retreatment management strategies when first-line tuberculosis therapy fails. Int J Tuberc Lung Dis **9:**421-429.
7. **Joseph P, Desai VB, Mohan NS, Fredrick JS, Ramachandran R, Raman B, Wares F, Ramachandran R, Thomas A.** 2011. Outcome of standardized treatment for patients with MDR-TB from Tamil Nadu, India. Indian J Med Res **133:**529-534.
8. **Ahuja SD, Ashkin D, Avendano M, Banerjee R, Bauer M, Bayona JN, Becerra MC, Benedetti A, Burgos M, Centis R, Chan ED, Chiang CY, Cox H, D'Ambrosio L, DeRiemer K, Dung NH, Enarson D, Falzon D, Flanagan K, Flood J, Garcia-Garcia ML, Gandhi N, Granich RM, Hollm-Delgado MG, Holtz TH, Iseman MD, Jarlsberg LG, Keshavjee S, Kim HR, Koh WJ, Lancaster J, Lange C, de Lange WC, Leimane V, Leung CC, Li J, Menzies D, Migliori GB, Mishustin SP, Mitnick CD, Narita M, O'Riordan P, Pai M, Palmero D, Park SK, Pasvol G, Pena J, Perez-Guzman C, Quelapio MI, Ponce-de-Leon A, et al.** 2012. Multidrug resistant pulmonary tuberculosis treatment regimens and patient outcomes: an individual patient data meta-analysis of 9,153 patients. PLoS Med **9:**e1001300. doi:10.1371/journal.pmed.1001300.
9. **Bernard C, Veziris N, Brossier F, Sougakoff W, Jarlier V, Robert J, Aubry A.** 2015. Molecular diagnosis of fluoroquinolone resistance in Mycobacterium tuberculosis. Antimicrob Agents Chemother **59:**1519-1524. doi:10.1128/aac.04058-14.
10. **Cui Z, Wang J, Lu J, Huang X, Zheng R, Hu Z.** 2013. Evaluation of methods for testing the susceptibility of clinical Mycobacterium tuberculosis isolates to pyrazinamide. J Clin Microbiol **51:**1374-1380. doi:10.1128/JCM.03197-12.
11. **Boehme CC, Nabeta P, Hillemann D, Nicol MP, Shenai S, Krapp F, Allen J, Tahirli R, Blakemore R, Rustomjee R, Milovic A, Jones M, O'Brien SM, Persing DH, Ruesch-Gerdes S, Gotuzzo E, Rodrigues C, Alland D, Perkins MD.** 2010. Rapid molecular detection of tuberculosis and rifampin resistance. N Engl J Med **363:**1005-1015. doi:10.1056/NEJMoa0907847.
12. **Kurbatova EV, Kaminski DA, Erokhin VV, Volchenkov GV, Andreevskaya SN, Chernousova LN, Demikhova OV, Ershova JV, Kaunetis NV, Kuznetsova TA, Larionova EE, Smirnova TG, Somova TR, Vasilieva IA, Vorobieva AV, Zolkina SS, Cegielski JP.** 2013. Performance of Cepheid (R) Xpert MTB/RIF (R) and TB-Biochip

(R) MDR in two regions of Russia with a high prevalence of drug-resistant tuberculosis. Eur J Clin Microbiol Infect Dis **32:**735-743. doi:10.1007/s10096-012-1798-0.

13. **Simons SO, van der Laan T, Mulder A, van Ingen J, Rigouts L, Dekhuijzen PN, Boeree MJ, van Soolingen D.** 2014. Rapid diagnosis of pyrazinamide-resistant multidrug-resistant tuberculosis using a molecular-based diagnostic algorithm. Clin Microbiol Infect **20:**1015-1020. doi:10.1111/1469-0691.12696
14. **Falzon D, Jaramillo E, Wares F, Zignol M, Floyd K, Raviglione MC.** 2013. Universal access to care for multidrug-resistant tuberculosis: an analysis of surveillance data. Lancet Infect Dis **13:**690-697. doi:10.1016/s1473-3099(13)70130-0.
15. **Smith SE, Kurbatova EV, Cavanaugh JS, Cegielski JP.** 2012. Global isoniazid resistance patterns in rifampin-resistant and rifampin-susceptible tuberculosis. Int J Tuberc Lung Dis **16:**203-205. doi:10.5588/ijtld.11.0445.
16. **Espinal MA, Kim SJ, Suarez PG, Kam KM, Khomenko AG, Migliori GB, Baez J, Kochi A, Dye C, Raviglione MC.** 2000. Standard short-course chemotherapy for drug-resistant tuberculosis: treatment outcomes in 6 countries. Jama **283:**2537-2545.
17. **Yee DP, Menzies D, Brassard P.** 2012. Clinical outcomes of pyrazinamide-monoresistant Mycobacterium tuberculosis in Quebec. Int J Tuberc Lung Dis **16:**604-609. doi:10.5588/ijtld.11.0376.
18. **Orenstein EW, Basu S, Shah NS, Andrews JR, Friedland GH, Moll AP, Gandhi NR, Galvani AP.** 2009. Treatment outcomes among patients with multidrug-resistant tuberculosis: systematic review and meta-analysis. Lancet Infect Dis **9:**153-161. doi:10.1016/S1473-3099(09)70041-6
19. **Falzon D, Gandhi N, Migliori GB, Sotgiu G, Cox HS, Holtz TH, Hollm-Delgado MG, Keshavjee S, DeRiemer K, Centis R, D'Ambrosio L, Lange CG, Bauer M, Menzies D, Collaborative Group for Meta-Analysis of Individual Patient Data in M-T.** 2013. Resistance to fluoroquinolones and second-line injectable drugs: impact on multidrug-resistant TB outcomes. Eur Respir J **42:**156-168. doi:10.1183/09031936.00134712.
20. **Menzies D, Benedetti A, Paydar A, Martin I, Royce S, Pai M, Vernon A, Lienhardt C, Burman W.** 2009. Effect of duration and intermittency of rifampin on tuberculosis treatment outcomes: a systematic review and meta-analysis. PLoS Med **6:**e1000146. doi:10.1371/journal.pmed.1000146.
21. **Menzies D, Benedetti A, Paydar A, Royce S, Madhukar P, Burman W, Vernon A, Lienhardt C.** 2009. Standardized treatment of active tuberculosis in patients with previous treatment and/or with mono-resistance to isoniazid: a systematic review and meta-analysis. PLoS Med **6:**e1000150.
22. **Lew W, Pai M, Oxlade O, Martin D, Menzies D.** 2008. Initial drug resistance and tuberculosis treatment outcomes: systematic review and meta-analysis. Ann Intern Med **149:**123-134.
23. **Gagneux S, Long CD, Small PM, Van T, Schoolnik GK, Bohannan BJ.** 2006. The competitive cost of antibiotic resistance in Mycobacterium tuberculosis. Science **312:**1944-1946. doi:10.1126/science.1124410.
24. **O'Sullivan DM, McHugh TD, Gillespie SH.** 2010. Mapping the fitness of Mycobacterium tuberculosis strains: a complex picture. J Med Microbiol **59:**1533-1535. doi:10.1099/jmm.0.019091-0.

25. **Salvatore PP, Becerra MC, Abel Zur Wiesch P, Hinkley T, Kaur D, Sloutsky A, Cohen T.** 2016. Fitness Costs of Drug Resistance Mutations in Multidrug-Resistant Mycobacterium tuberculosis: A Household-Based Case-Control Study. J Infect Dis **213:**149-155. doi:10.1093/infdis/jiv347.
26. **Sergeev R, Colijn C, Cohen T.** 2011. Models to understand the population-level impact of mixed strain M. tuberculosis infections. J Theor Biol **280:**88-100. doi:10.1016/j.jtbi.2011.04.011.
27. **Blower SM, McLean AR, Porco TC, Small PM, Hopewell PC, Sanchez MA, Moss AR.** 1995. The intrinsic transmission dynamics of tuberculosis epidemics. Nat Med **1:**815-821.
28. **Harries AD, Dye C.** 2006. Tuberculosis. Ann Trop Med Parasitol **100:**415-431. doi:10.1179/136485906X91477.
29. **World Health Organization.** 2010. Guidelines for treatment of tuberculosis. WHO, Geneva. http://www.who.int/tb/publications/2010/9789241547833/en/. Accessed June 29, 2015.
30. **Sutherland I, Svandova E, Radhakrishna S.** 1982. The development of clinical tuberculosis following infection with tubercle bacilli. 1. A theoretical model for the development of clinical tuberculosis following infection, linking from data on the risk of tuberculous infection and the incidence of clinical tuberculosis in the Netherlands. Tubercle **63:**255-268.
31. **Vynnycky E, Fine PE.** 1997. The natural history of tuberculosis: the implications of age-dependent risks of disease and the role of reinfection. Epidemiol Infect **119:**183-201.
32. **Behr MA, Warren SA, Salamon H, Hopewell PC, Ponce de Leon A, Daley CL, Small PM.** 1999. Transmission of Mycobacterium tuberculosis from patients smear-negative for acid-fast bacilli. Lancet **353:**444-449.
33. **Tiemersma EW, van der Werf MJ, Borgdorff MW, Williams BG, Nagelkerke NJ.** 2011. Natural history of tuberculosis: duration and fatality of untreated pulmonary tuberculosis in HIV negative patients: a systematic review. PLoS One **6:**e17601. doi:10.1371/journal.pone.0017601.
34. **Division UNP.** 2013. World Population Prospects: The 2012 Revision. United Nations, New York. http://data.un.org/Data.aspx?q=life+expectancy&d=PopDiv&f=variableID%3a68. Accessed June 29, 2015.
35. **Sunnaker M, Busetto AG, Numminen E, Corander J, Foll M, Dessimoz C.** 2013. Approximate Bayesian computation. PLoS Comput Biol **9:**e1002803. doi:10.1371/journal.pcbi.1002803.
36. **Franke MF, Becerra MC, Tierney DB, Rich ML, Bonilla C, Bayona J, McLaughlin MM, Mitnick CD.** 2015. Counting pyrazinamide in regimens for multidrug-resistant tuberculosis. Ann Am Thorac Soc **12:**674-679. doi:10.1513/AnnalsATS.201411-538OC.
37. **Slinker BK, Glantz SA.** 1985. Multiple regression for physiological data analysis: the problem of multicollinearity. Am J Physiol **249:**R1-12.
38. **Cao Y, Gillespie DT, Petzold LR.** 2007. Adaptive explicit-implicit tau-leaping method with automatic tau selection. J Chem Phys **126:**224101. doi:10.1063/1.2745299.
39. **World Health Organization.** 2015. Global Tuberculosis Report 2015. WHO, Geneva. http://www.who.int/tb/publications/global_report/en/. Accessed January 20, 2016.

40. **World Health Organization.** 2011. Towards universal access to diagnosis and treatment of multidrug-resistant and extensively drug-resistant tuberculosis by 2015: WHO progress report 2011. WHO, Geneva. http://www.who.int/tb/publications/2011/mdr_report_2011/en/. Accessed June 29, 2015.
41. **Pierre-Audigier C, Surcouf C, Cadet-Daniel V, Namouchi A, Heng S, Murray A, Guillard B, Gicquel B.** 2012. Fluoroquinolone and pyrazinamide resistance in multidrug-resistant tuberculosis. Int J Tuberc Lung Dis **16:**221-223, i-ii. doi:10.5588/ijtld.11.0266.
42. **Hannan MM, Desmond EP, Morlock GP, Mazurek GH, Crawford JT.** 2001. Pyrazinamide-monoresistant Mycobacterium tuberculosis in the United States. J Clin Microbiol **39:**647-650. doi:10.1128/jcm.39.2.647-650.2001.
43. **Djuretic T, Herbert J, Drobniewski F, Yates M, Smith EG, Magee JG, Williams R, Flanagan P, Watt B, Rayner A, Crowe M, Chadwick MV, Middleton AM, Watson JM.** 2002. Antibiotic resistant tuberculosis in the United Kingdom: 1993-1999. Thorax **57:**477-482.
44. **Umubyeyi AN, Rigouts L, Shamputa IC, Fissette K, Elkrim Y, de Rijk PW, Struelens MJ, Portaels F.** 2007. Limited fluoroquinolone resistance among Mycobacterium tuberculosis isolates from Rwanda: results of a national survey. J Antimicrob Chemother **59:**1031-1033. doi:10.1093/jac/dkm038.
45. **Ginsburg AS, Hooper N, Parrish N, Dooley KE, Dorman SE, Booth J, Diener-West M, Merz WG, Bishai WR, Sterling TR.** 2003. Fluoroquinolone resistance in patients with newly diagnosed tuberculosis. Clin Infect Dis **37:**1448-1452. doi:10.1086/379328.
